# Development and Evaluation of a Realistic Audio-Visual Multi-Talker Speech Comprehension Test

**DOI:** 10.1177/23312165261489985

**Published:** 2026-09-17

**Authors:** Xinyu Guo, Rebecca Holt, Kelly Miles, Ronny Kurniawan Ibrahim, Stefan Raufer, Peter Derleth, Joerg M. Buchholz

**Affiliations:** 1 Department of Linguistics, 336640 Macquarie University, Sydney, Australia; 2 Ecological Hearing Outcomes (ECHO) Laboratory, Macquarie University, Sydney, Australia; 3 Sonova AG, Research & Development, Stäfa, Switzerland

**Keywords:** realistic assessment, speech comprehension, listening effort, multi-talker conversation, audio-visual speech

## Abstract

Current speech comprehension assessments do not provide a reliable measure of listeners’ real-life speech comprehension ability, as they fail to capture the complexity and dynamic nature of real-world communicative scenarios. To better align laboratory assessment with real-world listening demands, we developed a new speech comprehension test using audio-visual recordings of spontaneous conversations between two talkers. The conversations were combined with babble noise at three different signal-to-noise ratios (SNRs) to vary task difficulty (Quiet, 0dB, and -5dB). Speech comprehension was assessed using a word monitoring paradigm, in which listeners were given a word category and responded to target words related to that category by pressing a hand-held button. Outcome measures included self-rated listening effort, hit rate, response time (RT), and head turn behavior. Thirty adults with normal hearing were tested. With decreasing SNR, self-reported listening effort increased, hit rate decreased, and RT increased. Participants predominantly oriented toward the active talker, with visual localization achieved through a combination of head and eye movements. Dynamic analyses around conversational floor transfers revealed that, listeners oriented earlier and more consistently toward the upcoming talker in more challenging conditions. None of the behavioral measures were significantly associated with listening effort ratings after accounting for SNR effects. Overall, our results demonstrate that the proposed test provides a sensitive measure of real-world speech comprehension and holds promise for assessing the impact of hearing loss and evaluating hearing device benefit in ecologically valid settings.

## Introduction

Conversation is the most common and essential listening scenario in everyday life (Pichora-Fuller et al., 2016; Smeds et al., 2020). Fundamental to participating in a conversation is speech comprehension, which represents a higher level of processing than hearing or listening alone (Kiessling et al., 2003). Hearing refers to the detection and encoding of acoustic information, while listening involves attending to and identifying speech signals. Speech comprehension further encompasses higher-level processing, including lexical access, semantic interpretation, integration of information across utterances, use of contextual and visual cues, prediction of upcoming content, and flexible allocation of attention between talkers. However, most widely used speech tests do not adequately reflect the demands of real-world speech comprehension, e.g., the Hearing in Noise Test (HINT) (Soli & Wong, 2008), Quick Speech-in-Noise (QuickSIN) test (Killion et al., 2004), and Arizona Biomedical (AzBio) sentence test (Spahr et al., 2012). These commonly used tests typically omit key ecological features of everyday conversation, including speech with varying vocal effort, dynamic conversational floor transfers, spontaneous disfluencies (e.g., hesitations and repairs), audio-visual speech cues, and the need to switch attention between multiple interacting talkers.

Assessing speech comprehension rather than speech intelligibility alone may also better capture the listening effort experienced by people with hearing loss in their daily life, who frequently report that, although they may understand what is being said, they must exert substantial effort to do so (Rönnberg et al., 2013). Listening effort is defined as the deliberate allocation of cognitive resources to overcome obstacles in goal-directed listening (Pichora-Fuller et al., 2016). In adverse listening conditions, listeners may therefore achieve successful speech understanding, but only at the cost of increased mental effort. This dimension of communication difficulty is not captured by current clinical speech assessments, even though elevated listening effort has been associated with listening-related fatigue, reduced participation, and poorer quality of life in people with hearing loss (Davis et al., 2021; Hughes et al., 2018).

The aim of this study was to develop a new, realistic audio-visual speech comprehension test capable of measuring real-life speech comprehension in noisy, multi-talker environments. The test employed a word monitoring paradigm: listeners were given a semantic category (i.e., topic) and asked to respond whenever they detected a word in the conversation that belonged to that category, thereby requiring them to relate recognized words to the ongoing conversational topic. The test provided measures of accuracy (hit rate), response time, and self-rated listening effort. While participants performed the comprehension task, we additionally recorded their head and eye movements to characterize how listeners allocate audiovisual attention, particularly during conversational floor transfers. Unlike the behavioral measures and subjective ratings, head- and gaze-orienting measures provide a continuous and time-resolved record of listener behavior during ongoing conversation and may therefore help identify implicit markers of listening difficulty relevant to the development of adaptive hearing devices. In the following paragraphs, we briefly review the literature on real-life speech comprehension and the limitations of current comprehension assessments, before turning to listening effort in multi target talker scenarios with a particular focus on head and eye movements.

### Real-Life Speech Comprehension

Hearing performance is commonly assessed using pure tone audiometry (PTA), and listening performance is often assessed using word or sentence recognition tests. These measures capture important aspects of speech perception and can engage cognitive processes, particularly in noisy conditions. However, there is currently no standard test specifically designed to assess speech comprehension in realistic conversational settings. Speech comprehension is defined here as the process by which a listener perceives, interprets, and understands spoken language over time, drawing on the integration of peripheral auditory, central auditory, and cognitive processing (Gordon-Salant et al., 2020). In adverse or complex listening situations, top-down cognitive processes help compensate for degraded or missing information, and speech comprehension performance may be influenced by individual listener-related factors such as hearing loss, age, and cognitive decline (Jayakody et al., 2025).

Speech comprehension is influenced not only by these listener-related factors but also by the characteristics of the listening scenario (Mattys et al., 2012). In real-world situations, conversations often involve multiple talkers, requiring listeners to continuously switch attention between several potential targets to follow the conversation. Incorporating such dynamic, multi-target talker scenarios into speech comprehension assessments is therefore important. Beyond increasing overall comprehension demands – and thus overall task difficulty – a listener’s behavior in these scenarios may also provide insight into specific abilities relevant to participating in conversations, such as predicting turn transitions and reallocating attention between multiple talkers.

In challenging listening conditions, listeners may not be able to rely on auditory information effectively. Visual cues such as lip movements, head nods, and gestures then become especially important for supporting speech intelligibility (Yuan et al., 2021). In these circumstances it is advantageous for listeners to predict conversational floor transfers – that is, when one talker will finish and the next will begin speaking (see Sørensen et al. (2021)). By correctly recognizing these floor transfers, listeners can orient their eyes and head toward the upcoming talker even before speech onset, facilitating more effective use of both auditory and visual information (Hadley & Culling, 2022). However, inaccurate or delayed floor transfer prediction may limit the ability to make use of these cues. Effective prediction of floor transfers is therefore likely a key mechanism supporting comprehension in dynamic multi-talker conversations.

When observing rather than participating in a conversation, as in a speech comprehension task, listeners are not constrained by social norms and may shift their gaze or head orientation toward an upcoming talker even before the current speaker finishes. This motion behavior - specifically the timing of eye and head movements - may therefore reflect their ability to predict floor transfers. In challenging listening conditions, prediction of floor transfers may become less accurate or delayed, as auditory cues signaling upcoming turn transitions may be degraded.

Overall, incorporating multi-talker conversations (as speech material) into speech comprehension assessments may better reflect real-world listening demands, which place greater cognitive demands on listeners than single-talker speech. Furthermore, listeners’ head and eye movements in response to floor transfers may provide a time-resolved index of how effectively they predict conversational turn transitions and reallocate audiovisual attention during ongoing speech comprehension.

### Limitations of Current Speech Comprehension Assessments

In previous studies, the most common method for assessing a listener’s speech comprehension performance has been to present a recorded story and then to ask the listener to answer questions about its content (e.g., Schneider et al., 2000; Sommers et al., 2011). Other studies have attempted to measure real-time responses. For example, Hafter et al. (2013), frequently paused a story so that listeners could answer short questions, and Best et al. (2016) asked participants to provide brief written responses on a question sheet while listening to stories or conversations. Although these methods capture certain aspects of speech comprehension, they either disrupt the flow of the story, require participants to look at a response sheet (thus interfering with any visual speech cues) or make it difficult to separate the influence of non-listening-related abilities (e.g., episodic memory, reading and writing while listening) from the listening task itself. As a result, these methods may not provide a sensitive measure of true speech comprehension performance.

Moreover, most comprehension assessments have used scripted and rehearsed speech as stimuli, which tends to be fluent and clearly articulated and therefore diverges substantially from naturally occurring spontaneous speech (Laan, 1997). Spontaneous speech typically contains disfluencies, interjections, paralinguistic vocalizations (e.g., laughter), and phonetic reduction (Ernestus & Warner, 2011), and is rarely well-articulated or well-structured. These natural features can affect both the perceptual processing of the speech signal and higher-level comprehension (Ernestus & Warner, 2011; Mattys et al., 2012). In addition, real-life conversations often exhibit short-term or long-term contextual structure centered around core topics or themes, which can further facilitate speech comprehension (Brown-Schmidt & Tanenhaus, 2008). Incorporating spontaneous speech materials into comprehension assessments is therefore valuable for increasing ecological validity and improving the prediction of real-world speech-comprehension ability.

To address these issues, Buchholz et al. (2022) developed a continuous speech comprehension test based on the category-monitoring paradigm described by Marslen-Wilson and Tyler (1975). In this test, listeners are given a topic and then presented with an unscripted conversation recording containing multiple “target” words that are semantically related to that topic. Listeners are cued to the upcoming semantic category and instructed to press a handheld button whenever they hear a word belonging to that category. Since participants do not know the specific target words before the trial, this task engages real-time lexical recognition and semantic categorization processes. Speech materials were taken from the realistic two-talker conversation recordings of Miles et al. (2020). Although the test uses a real-time response measure to reduce the influence of episodic memory and incorporates spontaneous speech to provide more realistic listening conditions, it still has certain limitations − most notably, a restricted set of five conversations or topics (and therefore a limited number of target words or scoring units) and the use of auditory-only stimuli.

In summary, current methods do not provide a sensitive and comprehensive measure of listeners’ real-world speech comprehension ability. It is therefore important to develop an assessment that utilizes naturalistic (spontaneous) audiovisual materials and incorporates real-time response measures that more directly reflect an individual’s speech comprehension ability. Such an assessment would offer more meaningful insights and could be used to diagnose individuals with speech comprehension deficits (e.g., due to hearing loss) as well as to evaluate the benefits of hearing interventions (e.g., hearing devices and related technologies).

### Ecological Measurement – Listening Effort

In real-life situations, some individuals – especially those with hearing loss – report that although they can understand what is being said in a conversation, they must exert considerable effort to follow it, particularly in noisy environments (Hughes et al., 2018; Rönnberg et al., 2013). This is because listeners must allocate additional cognitive resources to process degraded auditory input. In everyday conversations, however, multiple sources of information can support comprehension. Complementary acoustic, linguistic, and visual cues (e.g., lip movements and gestures) provide additional information that helps listeners compensate for degraded auditory signals (Wingfield et al., 2005). Visual speech information in particular has been shown to become increasingly beneficial when comprehension requires semantic integration of speech content (e.g., Fleming & Winn, 2025; Reisberg, 1987). Nevertheless, when listening conditions become sufficiently challenging, the cognitive resources required to maintain comprehension may increase substantially and can lead to increased perceived listening effort and fatigue (Pichora-Fuller et al., 2016).

Currently, a wide range of measures are used to assess listening effort. These can be categorized into physiological, subjective, and behavioral measures (Rudner et al., 2012). Physiological measures capture listeners’ involuntary biological responses to listening demands, including pupillometry, functional magnetic resonance imaging (fMRI), electroencephalography (EEG), and autonomic nervous system metrics such as heart rate and skin conductance. Subjective measures assess a listener’s perceived listening effort based on self-reports or judgments, such as the adaptive categorical listening effort scaling procedure (ACALES; Krueger et al., 2017). Behavioral measures use single- or dual-task paradigms to evaluate listeners’ responses to listening tasks, such as measuring response time or accuracy on a secondary task performed concurrently with listening (Houben et al., 2013).

Each type of measure has its advantages and limitations. Physiological measures can provide an objective index of listening effort but are difficult to implement in a realistic speech comprehension test. In contrast, subjective and behavioral measurements are relatively easy to implement. However, subjective measures are prone to individual differences in scale interpretation and response bias (Giuliani et al., 2021) while behavioral measures may reflect listening effort only indirectly. Although outcomes such as response time or task accuracy are associated with cognitive load, they do not provide a direct measure of the resources expended during listening (Alhanbali et al., 2019).

In the present study, we included both subjective and behavioral measures to assess listening effort in a realistic multi-talker speech comprehension test, specifically, self-rating scales and task performance measures (i.e., response accuracy and response time). Combining these two types of measures provides complementary information about listening effort: response time is widely used to reflect the cognitive demands of processing speech during the task, whereas subjective ratings capture listeners’ perceived effort when following the conversation.

### Ecological Measurement – Head and Eye Movements

In real-life listening situations, listeners may turn their head and eyes to maximize visual speech information (Hadley et al., 2021), improve the signal-to-noise ratio (SNR) through the acoustic head shadow effect (Grange et al., 2018), or support pro-social behavior (Cañigueral & Hamilton, 2019). Studies on listening to multi-talker conversations have shown that listeners generally orient their gaze toward the active talker, whereas head orientation typically undershoots gaze direction but still provides an approximate indication of which talker they are looking at (Hendrikse et al., 2018; Lu et al., 2021). Together, these findings indicate that combined head and eye movements are relevant for characterizing how listeners orient toward talkers and access visual information in audiovisual conversation.

In addition to absolute rotation angles (or look directions), the orienting behavior and timing of head and eye movements may provide an important metric for characterizing speech processing demands in multi-talker listening scenarios. Hadley and Culling (2022) used an interactive conversation paradigm between groups of three participants and recorded the head switches of each participant. Participants showed more head switches at lower noise levels than at higher noise levels, indicating that in more challenging listening conditions, prediction of floor transfers became less precise. Hidalgo et al. (2022) evaluated speech comprehension in normal-hearing participants by measuring their gaze switch timing while watching videos of two-talker conversations. They found that gaze switches were delayed under more degraded speech. Furthermore, participants with more anticipatory gaze behavior (i.e., shorter gaze switch delays) demonstrated better speech comprehension. Shiell et al. (2023) applied a similar two-talker speech comprehension task and found that increasing processing demands (i.e., decreasing SNR) delayed participants’ orienting toward the next talker during floor transfers. However, due to the experimental setup, head movements were negligible in this paradigm.

In summary, head and gaze movements play an important role in audio-visual speech comprehension in multi-talker listening situations. In particular, the timing of head and gaze switches provides a continuous, time-resolved behavioral index of how effectively listeners predict conversational floor transfers and allocate audio-visual attention. The relationship between listening difficulty and orienting behavior is, however, not straightforward, as several competing mechanisms may be at play. If background noise disrupts acoustic turn-end cues (e.g., prosodic or temporal boundary signals; Sørensen et al., 2024), listeners may delay orienting toward a new talker until sufficient auditory evidence accumulates. Similarly, increased cognitive load may delay the prediction of turn ends. However, adverse listening conditions also increase reliance on visual information both for speech processing as well as for predicting conversational floor transfers (Hadley et al., 2019; Russell & Harte, 2025), potentially causing listeners to orient earlier or more reliably toward the upcoming talker. The present study therefore evaluates how orienting behavior changes across listening conditions and whether it reflects delayed auditory and cognitive processing or increased strategic reliance on visual cues.

### The Present Study

The present exploratory study represents a first step toward the development of a more realistic audio-visual speech comprehension assessment. Our primary aim was to determine whether a test based on spontaneous multi-talker conversation is sensitive to controlled changes in listening difficulty and whether it can provide relevant behavioral measures indicative of real-world listening, including accuracy (hit rate), response time, self-rated listening effort, and head and gaze movements during conversational floor transfers.

The motivation for pursuing such measures is that everyday communication often requires listeners to follow dynamic, multi-talker interactions drawing on both auditory and visual information - demands that are only partially reflected in conventional speech tests. Such ecologically valid, spatially dynamic assessment is particularly important for evaluating hearing device benefit, as current speech enhancement algorithms are predominantly optimized for single-talker situations and may perform differently in complex multi-talker environments. The current study focuses on establishing the feasibility and sensitivity of the proposed assessment in normal-hearing listeners. Future work will apply the assessment to evaluate the effect of hearing loss on performance in complex, dynamic listening situations, as well as the benefit provided by hearing devices.

## Methods

### Participants

Thirty (30) participants (10 female, 20 male) between 18 and 31 years of age (mean age = 23.9 years, standard deviation = 3.2) participated in the study. All participants received pure tone audiometry (PTA) screening and had hearing thresholds less than or equal to 20 dB HL at 0.5, 1, 2, and 4 kHz for both ears. They spoke English as their first language and reported no general communication difficulties. All participants gave electronic consent to participate using REDCap (Harris et al., 2009, 2019). The study was approved by the Macquarie Ethics Committee.

### Speech Material

#### Realistic Audio-Visual Conversation Recordings

The audio-visual two-talker conversation recordings from Miles et al. (2020) were used for the development of the speech materials. The corpus consisted of 19 spontaneous (unscripted) conversations between two Australian English speakers (female and male). Each conversation was approximately six minutes long (∼2 hours in total) and was recorded in one of three realistic background noise conditions taken from Weisser et al. (2019) and presented via open headphones to elicit normal, raised, and loud vocal effort levels (ANSI, 1997). The background noise was not captured in the recordings and is therefore not audible in the conversation stimuli used in the present experiment. The introduction of realistic vocal efforts makes the speech material more realistic, as talkers increase their vocal effort when talking in background noise to help the listener differentiate the speech signal from the noise (Cipriano et al., 2017). The two talkers were given different topics for each recording. They were then instructed to speak naturally as they typically would in a casual social setting and not to “overact” or “play a character.” This resulted in natural variations in talking speed, turn-taking behavior, turn durations, and conversational overlaps such as those encountered in the real world.

The audio-visual recordings were made in an acoustically treated studio equipped with green screens and diffuse lighting. Recordings were captured using two separate, directional boom microphones and a two-camera cross setup. The audio recordings contained minor acoustic crosstalk, which was removed using the de-bleed function in RX10 (iZotope.com). Similarly, excessive hand movements occasionally caused one talker’s hand to appear in the other talker’s video. These instances were manually corrected in Adobe Premiere. Additionally, slow fluctuations in speech level were flattened in MATLAB using the envelope processing method described by Buchholz et al. (2022). Each individual recording and talker (channel) were then RMS-level normalized according to the method described in IEC 60268-16 (2011, Annex J) to exclude speech pauses and silent intervals.

#### Topics and Target Words Selection

The first author, as well as three experienced researchers (two linguistics and one audiologist) who spoke English as their first language, listened to the 19 two-talker conversation recordings described in the above section to select an initial set of topics and target words while also checking the quality of the associated videos. Two Australian English speakers from non-research backgrounds then confirmed the selection of topics and target words. Note that the topics were selected based on the availability of target words and did not necessarily correspond to any of the 19 conversation topics provided to the actors during the studio recordings.

Fifty-eight (58) short “snippets” were cut from the 19 conversation recordings. Each snippet was between 12 and 50 seconds long (27 seconds on average). The snippets covered 46 topics in total, with some topics occurring in more than one snippet. They each contained two to eight target words (3 on average), providing a total of 173 target words across the 58 snippets. The target words had mean duration of 0.55 seconds (standard deviation = 0.31 seconds) and comprised of 2.42 syllables length (standard deviation = 1.63) and 6.43 phonemes (standard deviation = 3.94) on average. A list of the topics and associated target words can be found in the Appendix (Table 1).

#### Floor Transfer Coding

A floor transfer refers to the conversational transition from one talker to another. To characterize the floor transfer behavior of the two target talkers in the speech materials, their floor transfers were manually labeled for all 58 recording snippets following the procedure described by Sørensen et al. (2021). Thereby, the time stamp of a floor transfer was defined by the onset time of the new talker’s speech, as illustrated in Figure 1.

**Figure 1.**
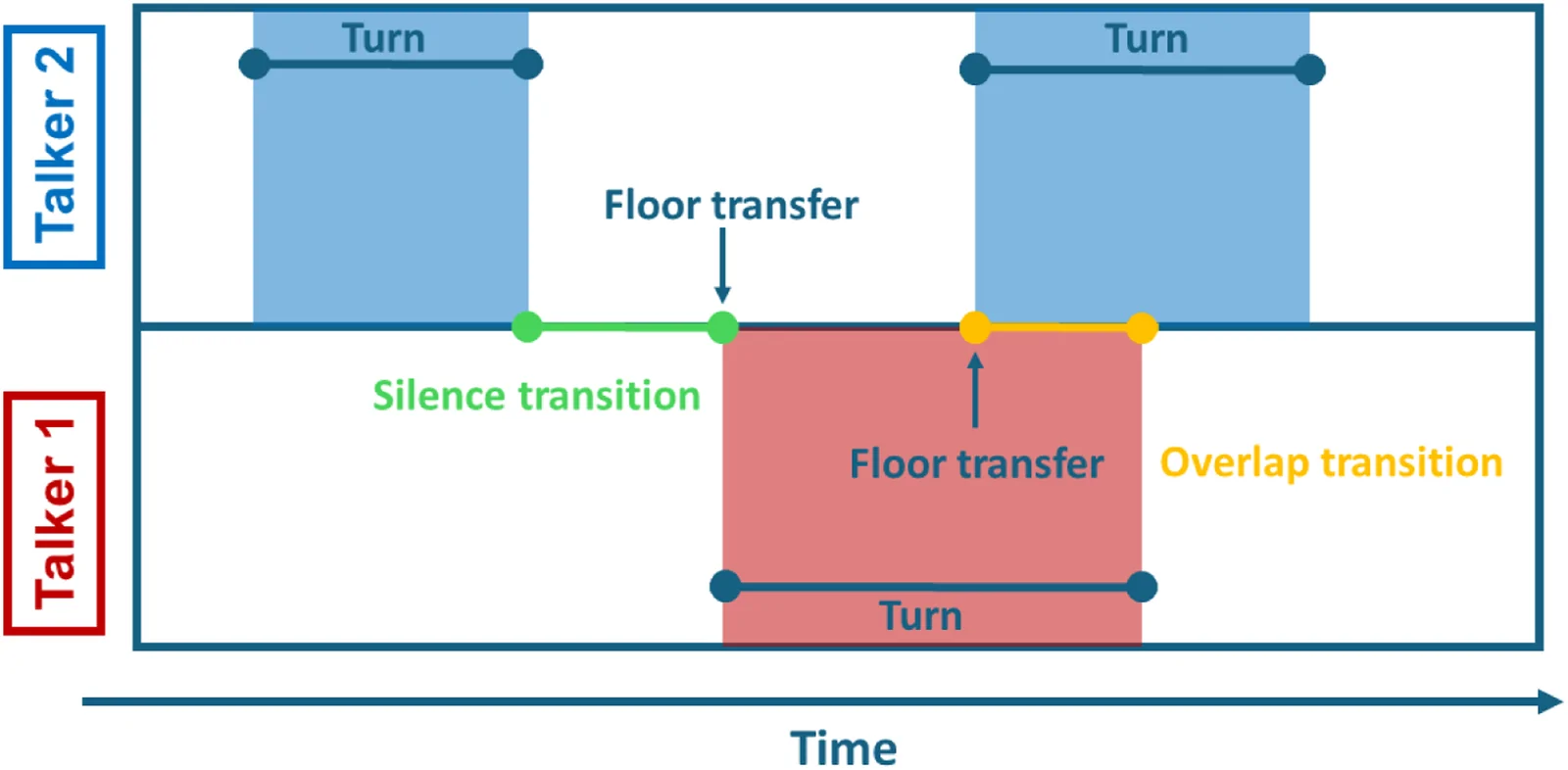
Schematic diagram illustrating the two types of conversational floor transfers

The speech materials included a total of 123 floor transfers. Ninety-two (92) were silent transfers where the speech of the first talker ended before the new talker started. In 31 transfers, there was an overlap between the speech of the two talkers during the turn. Figure 2 shows a histogram of the transition duration for all 123 floor transfers, where negative values indicate a speech overlap between talkers.

**Figure 2.**
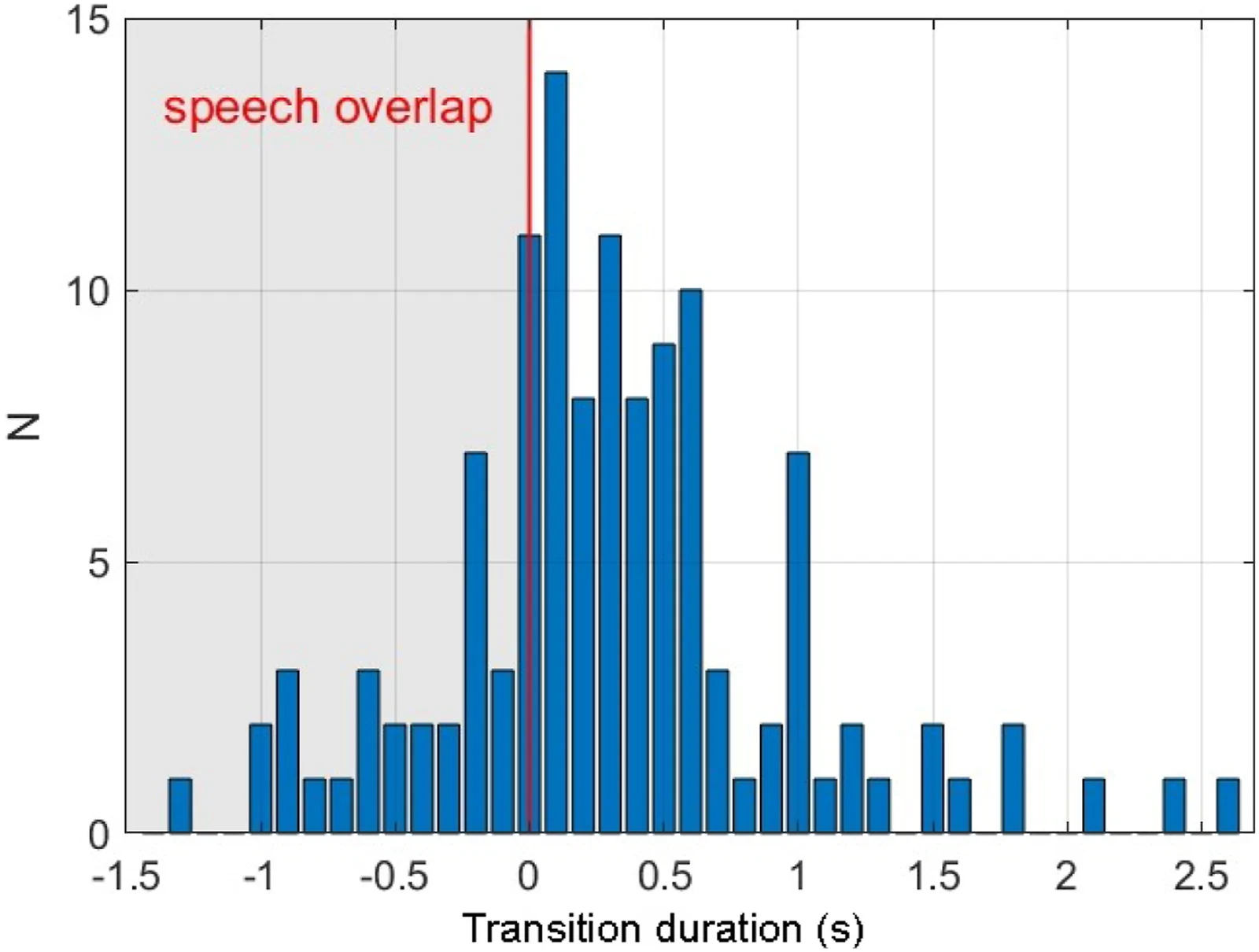
Histogram of the conversation transition duration between the first and second talker during the 123 floor transfers. A negative value indicates a talker overlap

### Experiment Setup and Stimuli

The study was conducted in the anechoic chamber of the Australian Hearing Hub using a spherical (3D) array of Tannoy V8 loudspeakers and an acoustically transparent cylindrical 200° screen. The placement of the 41 loudspeakers is shown in Figure 3. However, only six of the loudspeakers in the horizontal ring were used for stimulus presentation: Speaker 3 (right 45°) and Speaker 15 (left 45°) presented the target speech conversation materials; Speaker 1 (front 0°), Speaker 5 (right 90°), Speaker 9 (back 180°), and Speaker 13 (left 90°) presented the background noise. The background noise consisted of 32-talker speech babble, created by presenting the same 8-talker (4 male, 4 female) speech recording from each of the four loudspeakers with different time delays. The 8-talker speech babble recording was taken from Keidser et al. (2002) and was spectrally equalized to match the long-term average spectrum of the combined target speech material (i.e., combined across the 58 conversation snippets and the two talkers).

**Figure 3.**
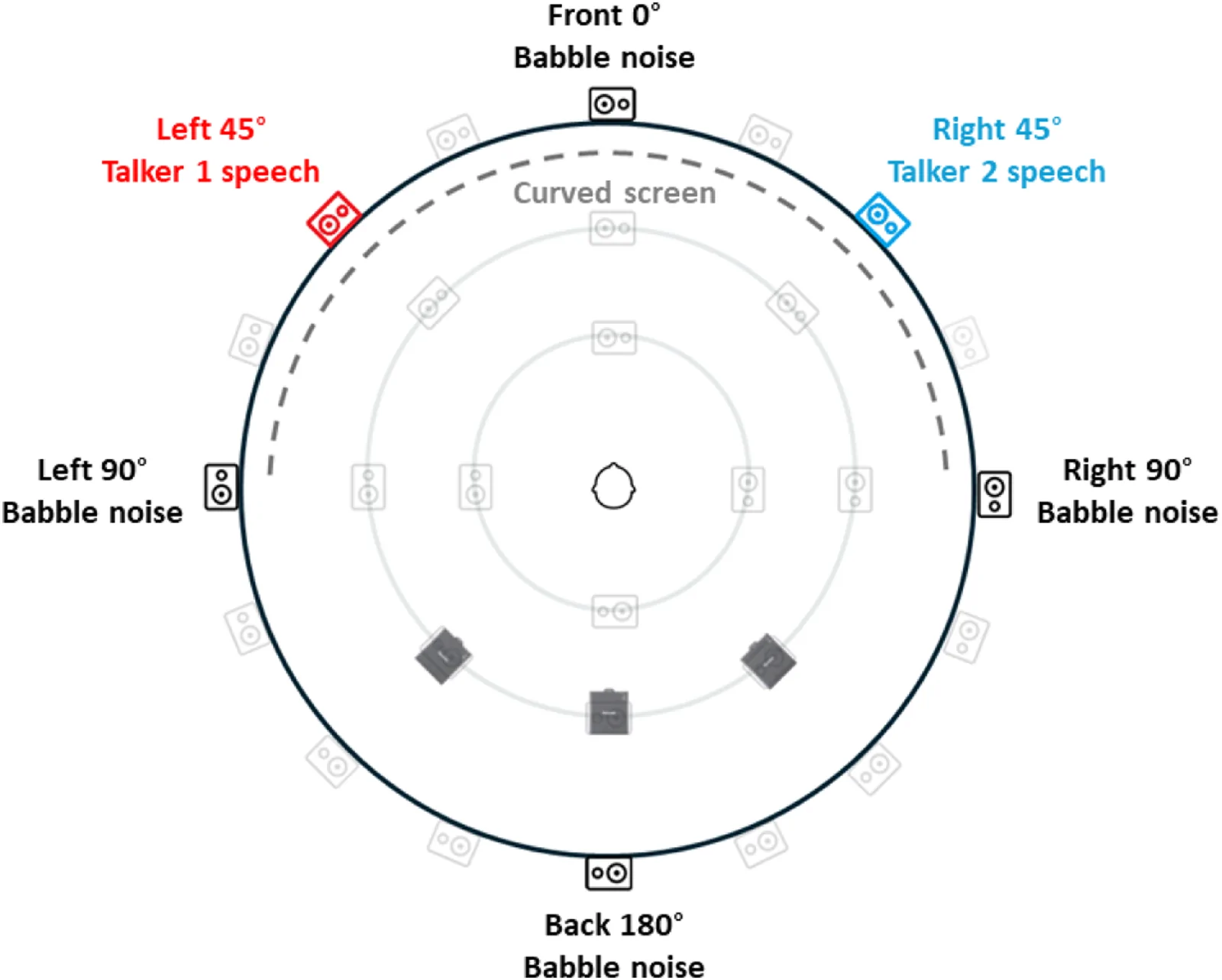
Top view of the 41-channel 3D speaker array and 200° curved screen. Irrelevant loudspeakers are semi-transparent

The speech of each of the two target talkers was always presented at a fixed sound pressure level of 65 dB-SPL (excluding pauses and silence). The level of the 32-talker babble noise was set to 60, 65, or -∞ dB SPL to provide three different SNRs of -5 dB, 0 dB, and ∞ dB (quiet).

The target speech conversation videos were chroma-keyed, and the individual talkers were positioned at -45° and + 45° on the 200° screen (see Figure 4), with their faces aligned with the corresponding loudspeakers. The original conversations were recorded with a two-camera cross setup in which the two talkers faced each other. When rendered at ±45°, the talkers therefore appear directed toward a common central point rather than toward each other. This arrangement was intended to simulate an observational perspective as if the participant were a third person in the conversation. The output video was rendered against a black background using an H.264 codec and mp4 container at a resolution of 5750x1200 pixels. The videos were projected onto the screen by three Panasonic PTRZ660 video projectors controlled via SimVisuals software.

**Figure 4.**
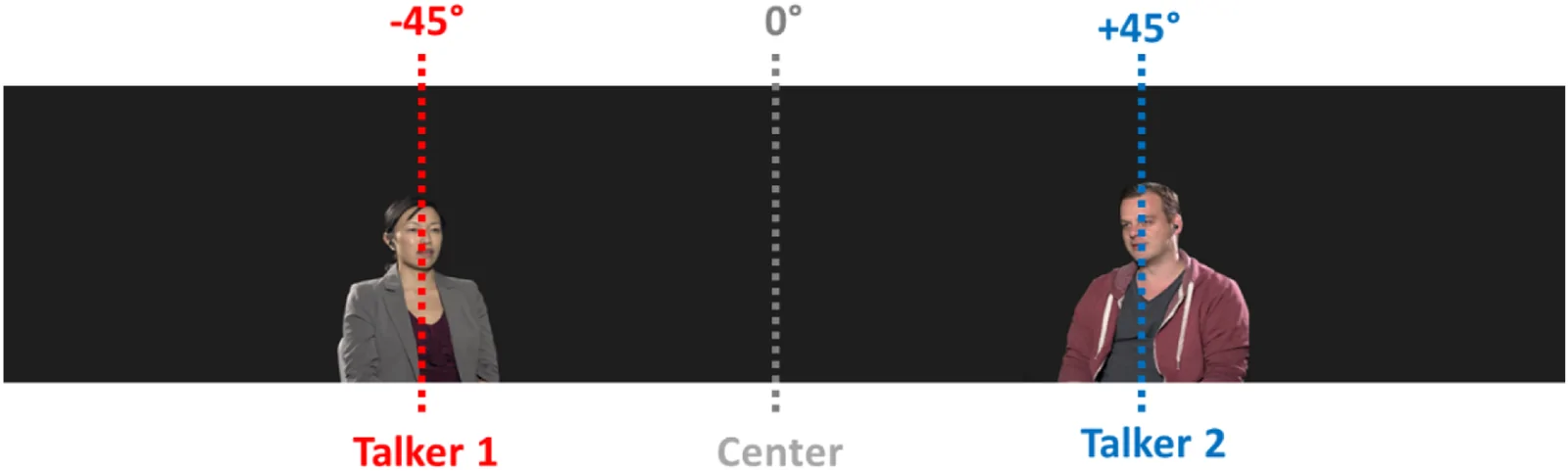
The placement of the two talkers on the curved screen (unrolled): talker 1 (female) was placed at left 45°, and talker 2 (male) was placed at right 45°

The audio-visual stimuli were controlled by test software developed in Matlab and played back using MaxMSP and Jitter (Cycling ’74) via Open Sound Control (OSC) commands. The test software ran on a desktop computer located outside the anechoic chamber and was operated by the test conductor, who communicated with the test person inside the chamber through an audio-visual intercom. The test computer was equipped with an RME MADI PCIe sound card connected to the 41 loudspeakers through two RME M-32 32-channel MADI-to-analog converters and eleven 4-channel Yamaha XM4080 power amplifiers. The audio-visual stimuli were presented at 50 frames per second with an audio sampling rate of 48 kHz.

The participants were positioned as shown in Figure 3 with their heads located at the center of the loudspeaker array. Head movements were tracked using a Vicon motion tracking system consisting of six infrared cameras mounted on the loudspeaker-array structure and connected to MX Ultranet hardware for data acquisition and synchronization. Eye movements were monitored with Tobii Pro 2 glasses fitted with six reflectors and integrated into the Vicon (MX Ultranet) system using an integration kit. The MX Ultranet hardware also received input from a handheld push button used for recording participant responses, as well as an audio trigger from the stimulus playback computer (embedded as an additional channel to the audio stimuli). This trigger channel was used to synchronize stimulus playback with the recorded button-press, head-tracking, and eye-tracking data. Target word and floor transfer times were predefined within the speech materials. These reference times enabled calculation of button-press latency and head-switch timing relative to each target word and floor transfer. The data acquisition system was controlled from a separate computer outside the anechoic chamber. Data were sampled at 100 Hz for the Vicon system and at 50 Hz for the Tobii glasses.

### Stimulus Randomization

The speech conversation materials were separated into 4 training trials and 54 test trials. To counterbalance the 54 test trials across the three SNR conditions, participants were grouped into sets of three, such that across each set, each conversation snippet was presented once at each SNR. Because each participant could listen to each snippet only once, this grouping allowed the same snippet to be tested across all three SNR conditions at the group level. The first two training trials were presented in quiet, followed by one training trial at 0 dB SNR and one at −5 dB SNR. For each participant, the assigned test trials were presented in randomized order, such that the three SNR conditions were intermixed rather than presented in separate blocks. In total, 30 individuals were tested in 10 sets of 3 participants.

Note that the original conversation recordings involved different vocal effort levels, whereas playback SNR was the manipulated factor in the present experiment. Snippets of different vocal effort levels were included to provide a sufficiently large and representative set of conversation materials. However, the limited number of available snippets neither allowed us to systematically match vocal effort with playback SNR, nor allowed us to include it as an additional experimental factor. Accordingly, the analysis focused on evaluating sensitivity to the experimentally controlled SNR conditions, which was the primary aim of the present exploratory study.

### Test Procedure

For each trial, participants were first presented with the written topic and three example target words projected onto the video screen, followed by audio-visual presentation of the corresponding conversation snippet. The participants pressed the hand-held response button whenever they heard a word that was semantically related to the topic (e.g., the word “rent” for the topic “expenses”, the word “Europe” for the topic “location”, or the word “cousins” for the topic “relatives”). The example target words were not presented in the test conversations. While performing this speech comprehension task, their eye and head movements were captured by the Tobii eye-tracking glasses and the Vicon infrared camera system. After each conversation was played, they verbally reported their perceived listening effort on a 7-point rating scale with descriptors (Krueger et al., 2022), which was then entered into the test software by the test conductor. The ratings were 1: no effort, 2: very little effort, 3: little effort, 4: moderate effort, 5: considerable effort, 6: very much effort, 7: extreme effort, and only noise. Before the experiment started, the test conductor verbally explained the concept of listening effort and instructed participants to rate based on the effort required to understand the individual conversation rather than on the task difficulty.

### Data Processing

#### Hit Rate

The hit rate was derived from the button press responses and calculated by the ratio between the total number of correct responses and the total number of presented target words, i.e.:

(1) $$Hit\;Rate=\frac{N_{correct\;responses}}{N_{presented\;target\;words}}\times 100\%$$

Similar to Buchholz et al. (2022), responses collected within a 3-second time window after the target word onsets were considered hits. After examining the response time distribution across all 30 participants, this window was further restricted to 0.3-2 seconds to better capture the expected range of human response times (see supplementary material, Figure 10).

Note that listeners rarely responded outside the designated response windows, and when they did, such responses were typically still semantically related to the conversation topic rather than random button presses. For instance, participants might have incorrectly responded to “baby towel” as a target in the family member category. As false alarms were not well defined in the present paradigm, they were excluded from the analysis.

#### Response Time

Response time (RT) was calculated for each hit, defined as the time difference between the participant’s absolute button press time and the onset time of the presented target word, i.e.:

(2) $$RT=T_{button\;press}-T_{target\;word\;onset}$$

If the target word contained multiple words (e.g., Cross City Tunnel), the onset time of the first word was considered as reference (e.g., Cross). Where multiple button presses were registered in the 0.3 to 2 seconds time window, only the first response was considered for the RT calculation.

#### Head Movement

The Vicon infrared motion-tracking system tracked the participants’ head movements using six reflective markers that were attached to the Tobii eye-tracking glasses. This provided the head azimuth angle α (as a function of time), which directly refers to the speaker array coordinate system and maps onto the 200° widescreen from -100° (left) to +100° (right) and 0° directly in front of the participant (Figure 5). The head movement data was sampled at 100 Hz.

**Figure 5.**
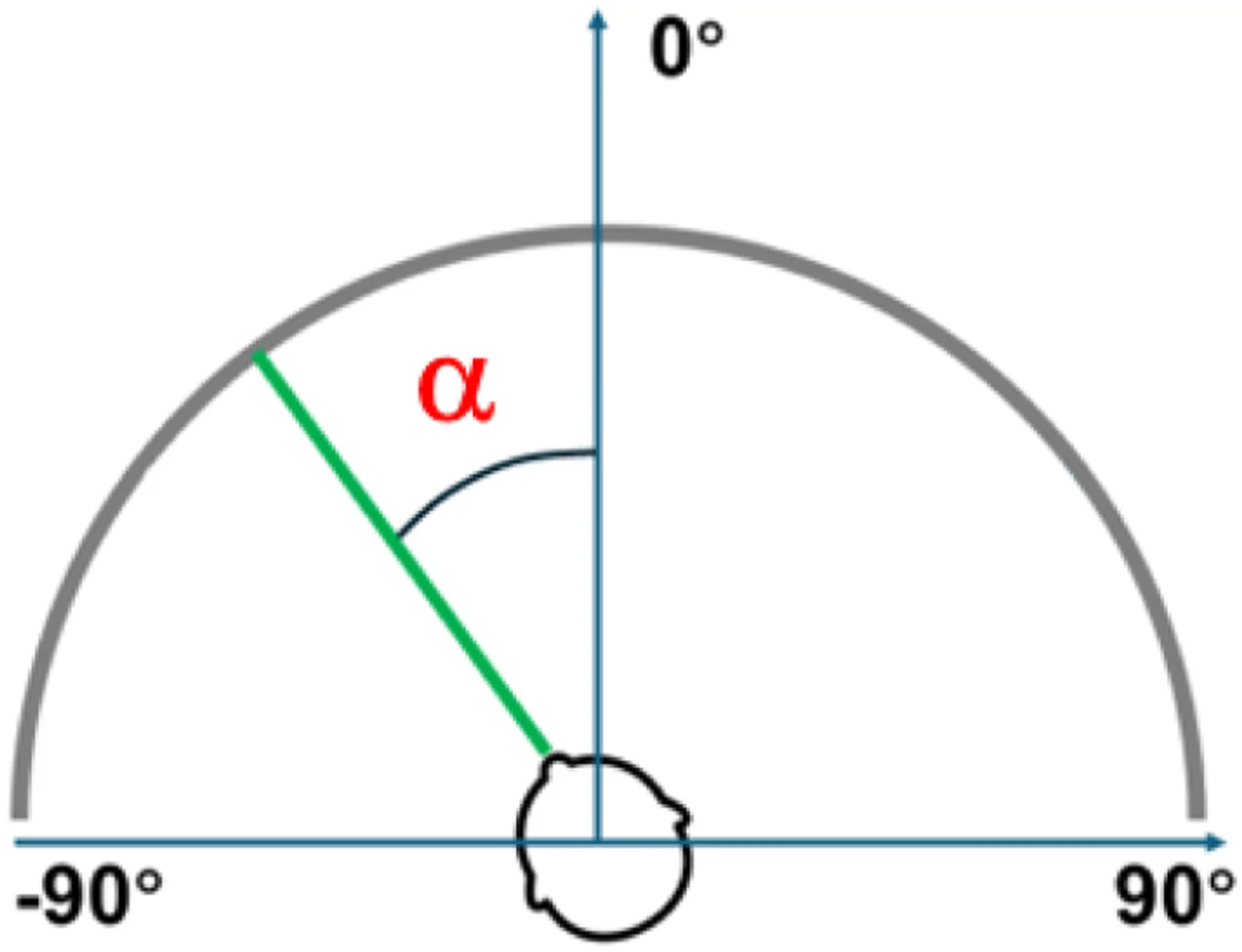
Head azimuth angle α

The head switch time (*T*_head switch_) refers to the time at which the participant’s head switches over from attending to one target talker to the other target talker. As participants’ head movements were not necessarily symmetric around the front (0°) direction, we first applied a k-means clustering algorithm to cluster their individual head-azimuth angle data into attending to the “left” and “right” target talkers for each of the 58 conversation snippets (Shiell et al., 2023). We then defined the “switch-over” (or reference) azimuth angle as the midpoint (or boundary) between these two clusters. The head switch time was then defined as the time when the head azimuth angle α crossed this reference angle, which for the specific case of symmetric head movements (i.e., a reference azimuth angle of 0°) is illustrated in Figure 6. The time difference between the head switch time and the floor transfer time (*T*_floor transfer_) is then defined as the head switch delay, i.e.,

(3) $$\tau_{head\;switch}=T_{head\;switch}-T_{floor\;transfer}$$

**Figure 6.**
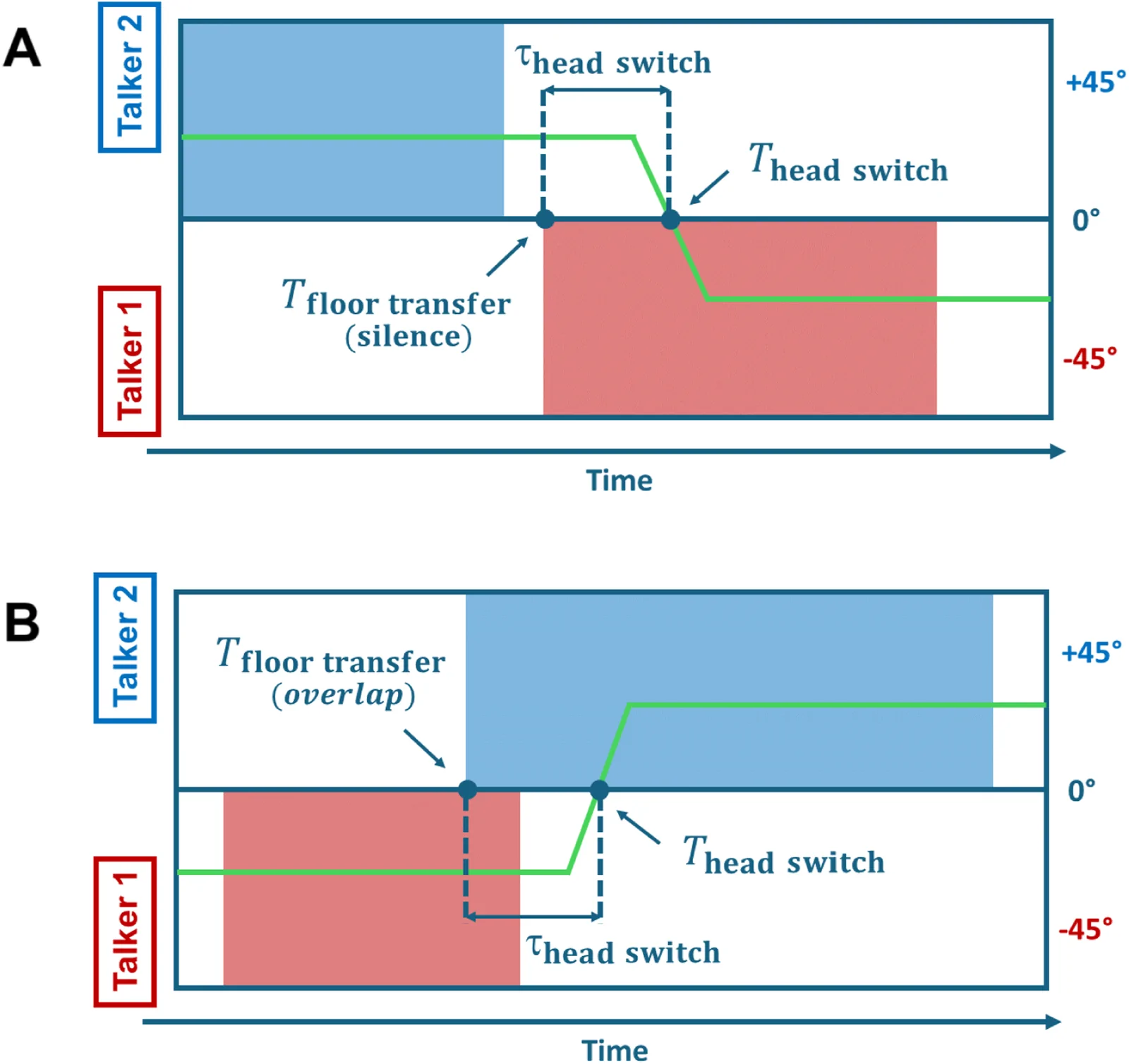
Illustration of the head switch delay derivation. The green line refers to the head azimuth angle. (a) head switch delay during silence transfer. (b) head switch delay during overlap transfer

Even though the “idealized” head turn pattern shown in Figure 6 can be observed across many test conditions, the head turn behavior is not always clearly defined. This is because the stimuli comprised natural conversations in which floor transfers are embedded within a broad range of multimodal interactions, including back-channeling, affirmations, gestures, head nodding, and overlapping speech. This results in complex head-turn behavior, including partial turns around floor transfers, brief orienting movements toward an anticipated next talker that are subsequently reversed, and occasional rapid switching between the two talkers when the floor transfer is ambiguous or is not fully developed. For a more robust derivation of the head switch delay, we used the k-means cluster boundaries to convert the absolute azimuth-angle data into binary values, with 1 indicating that the participant was looking toward the new talker and 0 indicating that they were looking toward the previous talker. The quality of the clustering was visually assessed for each participant and conversation snippet. This transformation allowed us to derive the probability (or odds) that a participant looked toward the new talker as a function of time relative to a floor transfer and enabled more robust data analyses such as logistic regression.

#### Gaze Movement

The Tobii eye-tracking glasses provided the azimuth angle of the point on the video screen at which the participant’s gaze was directed, relative to their head orientation. The gaze movement data was sampled at 50 Hz. In principle, the gaze data could be analyzed in the same way as the head-motion data (see above). However, we observed substantial data loss around floor transfers, likely due to extensive head movements. Because gaze and head movements are highly correlated, we decided not to include a temporal analysis of participants’ gaze behavior around floor transfers. The gaze data were only used descriptively to assess the participants’ overall gaze-direction distributions across conversation snippets and noise conditions.

### Statistical Analysis

All data and signal analyses were conducted using MATLAB (Version R2025b, The MathWorks Inc.) and R (R Core Team 2025). The statistical analyses followed a two-stage analytical approach. In the first stage, the individual outcome measures were analyzed to determine whether they were sensitive to controlled changes in listening difficulty (i.e., SNR). In the second stage, follow-up models were fitted to explore whether the different outcome measures were associated with listening effort, measured either subjectively via ratings or behaviorally via response time.

In the first stage, listening-effort ratings were analyzed using a cumulative link mixed model (CLMM) with a logit link, treating the 7-point effort ratings as an ordinal dependent variable, with SNR as a fixed effect and participant (N = 30) and conversation snippet (N = 54) as random intercepts. Hit rate data were analyzed using a generalized linear mixed-effects model (GLMM) with a binomial distribution and logit link, with SNR as a fixed effect and participant and target word (N = 162) as random intercepts. RT data were analyzed using a GLMM with an inverse Gaussian distribution and log link, following Lo and Andrews (2015), with SNR as a fixed effect and participant and target word as random intercepts.

For the effort rating, hit rate, and RT analyses, Helmert contrasts (Brehm & Alday, 2022) were used to compare the quiet condition with the average of the two noise conditions and to compare the two noise conditions directly. To maintain a comparable, parsimonious, and stable modeling framework across these three outcome measures, the primary models used random-intercept structures rather than random slopes.

For the head-turn analysis, sigmoid functions were fitted separately for each participant and SNR condition to characterize the probability of looking toward the new talker as a function of time relative to floor transfer. The fit used four derived parameters: slope (*k*), time shift (*τ*), minimum probability (*p*_*min*_), and maximum probability (*p*_*max*_). Additionally, based on the derived parameters, the time (*τ*_*50*_) at a probability of p=0.5 was calculated for each sigmoid function as an estimate of the head-turn delay. Four cases resulted in rather poor fits (R^2^ < 0.9) and were excluded from the analysis. These parameters were then analyzed in separate linear mixed-effects (LME) models with SNR as fixed effect and participant as random intercept. In addition, the binary head-turn time-series data were analyzed using a logistic mixed-effects model with time relative to floor transfer and SNR as fixed effects, and participant and floor transfer event (N = 117) as random intercepts.

For the second stage association analyses, all outcome measures were aggregated to one value per participant for each SNR condition. This was because the individual outcome measures varied in their item-level grouping factors, with listening-effort ratings obtained at the conversation-snippet level (N = 54), hit rate and RT at the target-word level (N = 162), and head-turn behavior measures at the floor transfer level (N = 117).

To explore associations between subjectively-assessed perceived listening effort and the different behavioral outcome measures, an LME model was fitted with listening-effort rating as the dependent variable, participant as a random intercept, and SNR, hit rate, RT (log-transformed to address the skewed distribution of the RT data), head-switch delay (as estimated by the *τ*_*50*_ parameter), and maximum probability of looking at the new talker (as estimated by the *p*_*max*_ parameter) as fixed effects. Hit rate and RT were included as speech comprehension performance measures, and *τ*_*50*_ and *p*_*max*_ were included as directly interpretable indices of head-turn behavior reflecting the timing of orientation and the extent to which listeners oriented toward the new talker.

To explore whether behaviorally-assessed listening effort (i.e., RT) was associated with head-turn behavior, a separate LME model was fitted with log-transformed RT as the dependent variable, participant as a random intercept, and SNR, head-switch delay, and maximum probability of looking at the new talker as fixed effects. Hit rate was omitted from this analysis, as RT can only be derived for correctly identified target words (i.e., hits), making RT non-independent of hit rate.

## Results

### Perceived Listening Effort, Hit Rate, and RT

Figure 7 shows boxplots of the main outcome measures as a function of SNR. Average effort ratings (a), hit rate (b), and average RT (c) were derived separately for each participant and SNR across all relevant test conditions. The median listening effort rating was 1.1 in quiet, 2.5 at 0 dB SNR, and 3.8 at −5 dB SNR. The median hit rate was 84.7% in quiet, 82.9% at 0 dB SNR, and 75.4% at −5 dB SNR. The median RT was 0.90s in quiet, 0.89s at 0 dB SNR, and 0.93s at −5 dB SNR. For all three measures, the Helmert contrast was significant between both quiet vs. noise and the two noise conditions (*p*<0.001).

**Figure 7.**
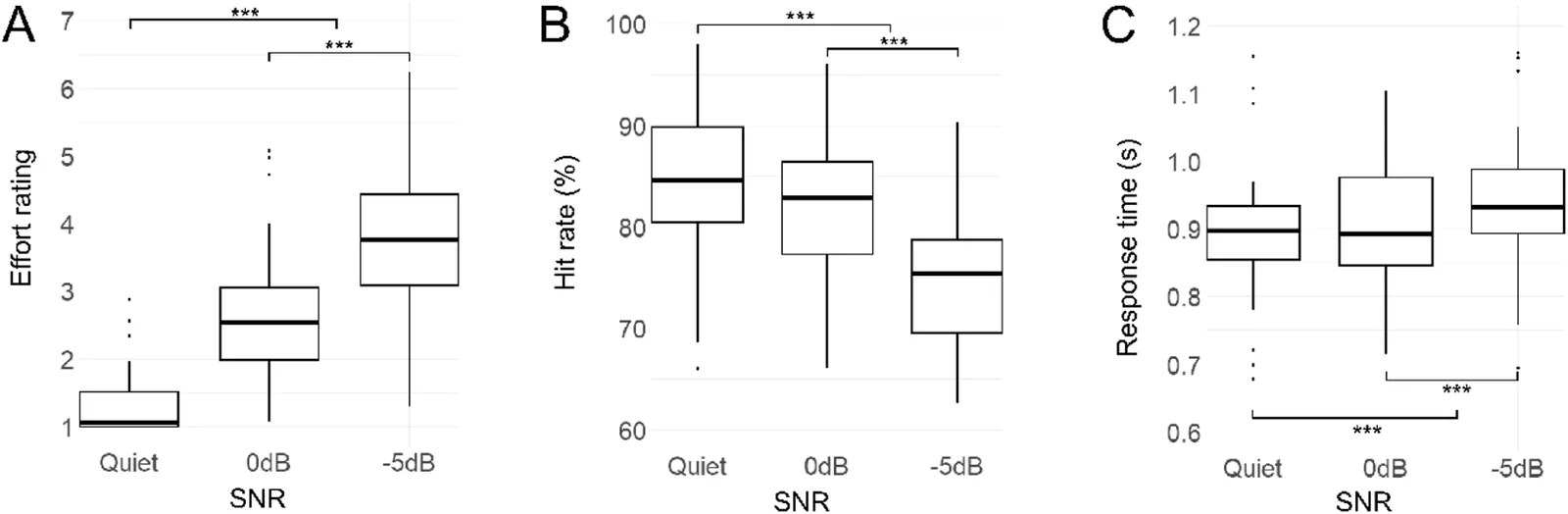
(a) Boxplots of listening effort rating as a function of SNR. (b) Boxplots of hit rate as a function of SNR. (c) Boxplots of RT as a function of SNR

### Head and Eye Movement

#### Histograms

Figure 8(a) shows participant-level heatmaps of head azimuth angle distributions for the 30 participants, derived across the 54 test trials and three SNR conditions (using a 2.5° bin width). For each participant, the counts in each azimuth bin were divided by the total number of valid samples for that participant. The color bar shows the ratio of samples in each azimuth bin for each participant. An azimuth angle of –45° corresponds to the position of talker 1, 0° represents the frontal position, and +45° corresponds to the position of talker 2. Most histograms display two distinct peaks pointing toward the two talkers, although the exact peak locations vary considerably across participants. On average, the peaks occur at approximately –12.5° and +12.5°, indicating that participants turned their heads only partially toward the target talkers at ±45°. Since we did not observe any noticeable differences in the overall distributions between SNR levels, the histograms in Figure 8 were averaged across the three SNR conditions.

**Figure 8.**
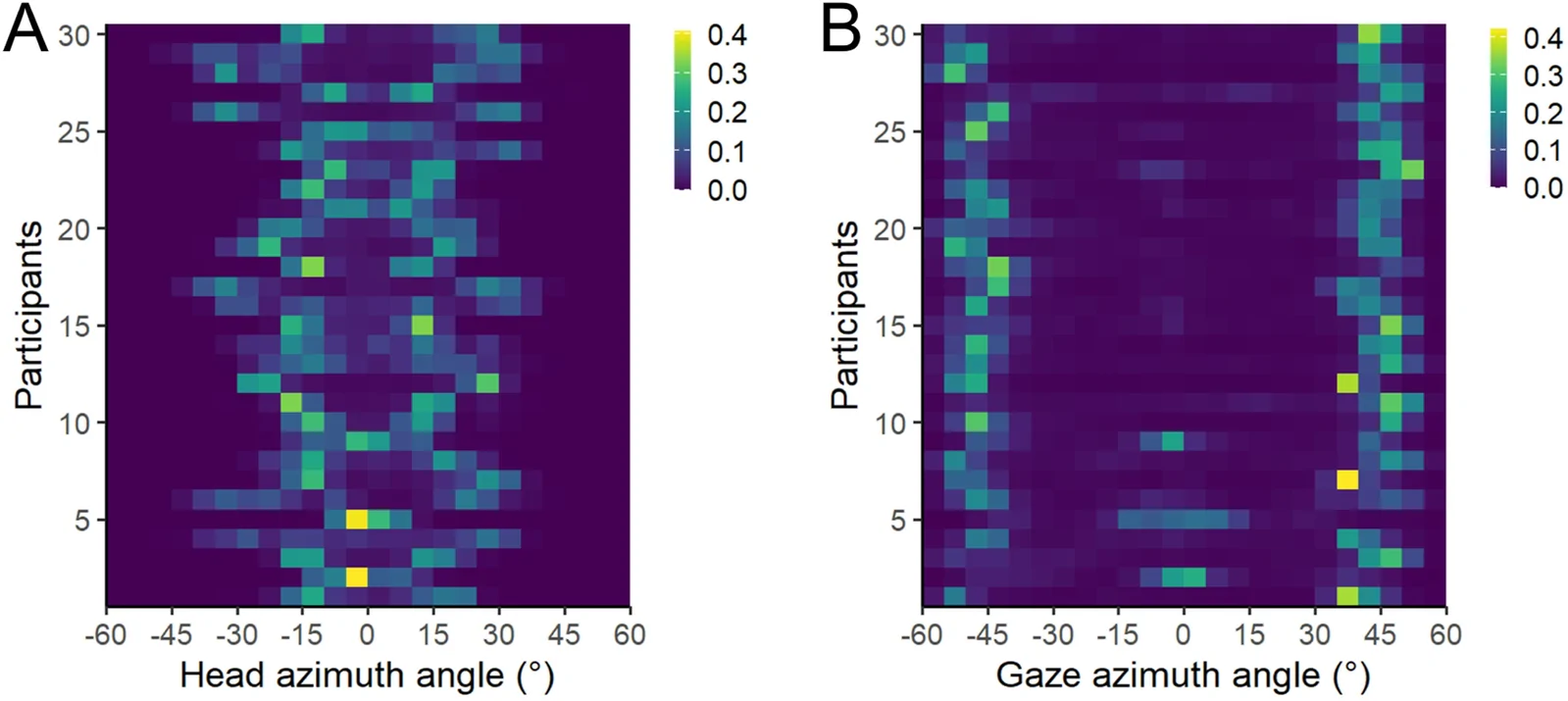
(a) Heatmap histograms of absolute head azimuth angles with participant as parameter. (b) Heatmap histograms of absolute gaze azimuth angles (i.e., head azimuth angles plus head-related gaze azimuth angles) with participant as parameter. The color scale represents the number of samples in each azimuth bin normalized by the total number of valid samples for each participant

Figure 8(b) shows participant-level heatmaps of absolute gaze-azimuth angle distributions (i.e., head azimuth plus head-relative gaze azimuth) for the 30 participants, derived across all 54 test trials and the three SNR conditions (with a bin width of 2.5°). The two histogram peaks now occur at approximately –47.5° and +47.5°, indicating that participants primarily directed their gaze toward the target talkers located at ±45°. Again, the SNR did not appear to affect the distribution and was therefore omitted. Notably, the histograms also show that three participants focused on the center of the screen rather than on the talker positions.

#### Dynamic Head Behavior Around Floor Transfers

To examine the dynamic head-turn behavior around floor transfers, the binary representation of the head turn data (see Methods section) was analyzed. For most participants, the k-means clustering clearly separated centroids corresponding to leftward and rightward head turns, and the midpoint between these centroids was close to 0°. There were three participants who made very little head movement, their two centroids were close to 0° and therefore less distinct. Examples of distinct clustering and less distinct clustering are shown in the supplementary materials (Figure 11). However, excluding the three participants did not affect the overall results. Figure 9 shows the probability of a participant looking at the new talker as a function of time (relative to the floor transfer) computed separately for the three SNR conditions, and pooled across all participants and conversational materials. The resulting curves reveal a substantial increase in the probability of looking toward the new talker after the floor transfer onset, rising from about p ≈ 0.1 to nearly p ≈ 0.9. The time at which each curve crosses p = 0.5 was considered an estimate of the head-switch delay, which was 0.89 s in quiet, 0.87 s at 0 dB SNR, and 0.81 s at −5 dB SNR.

**Figure 9.**
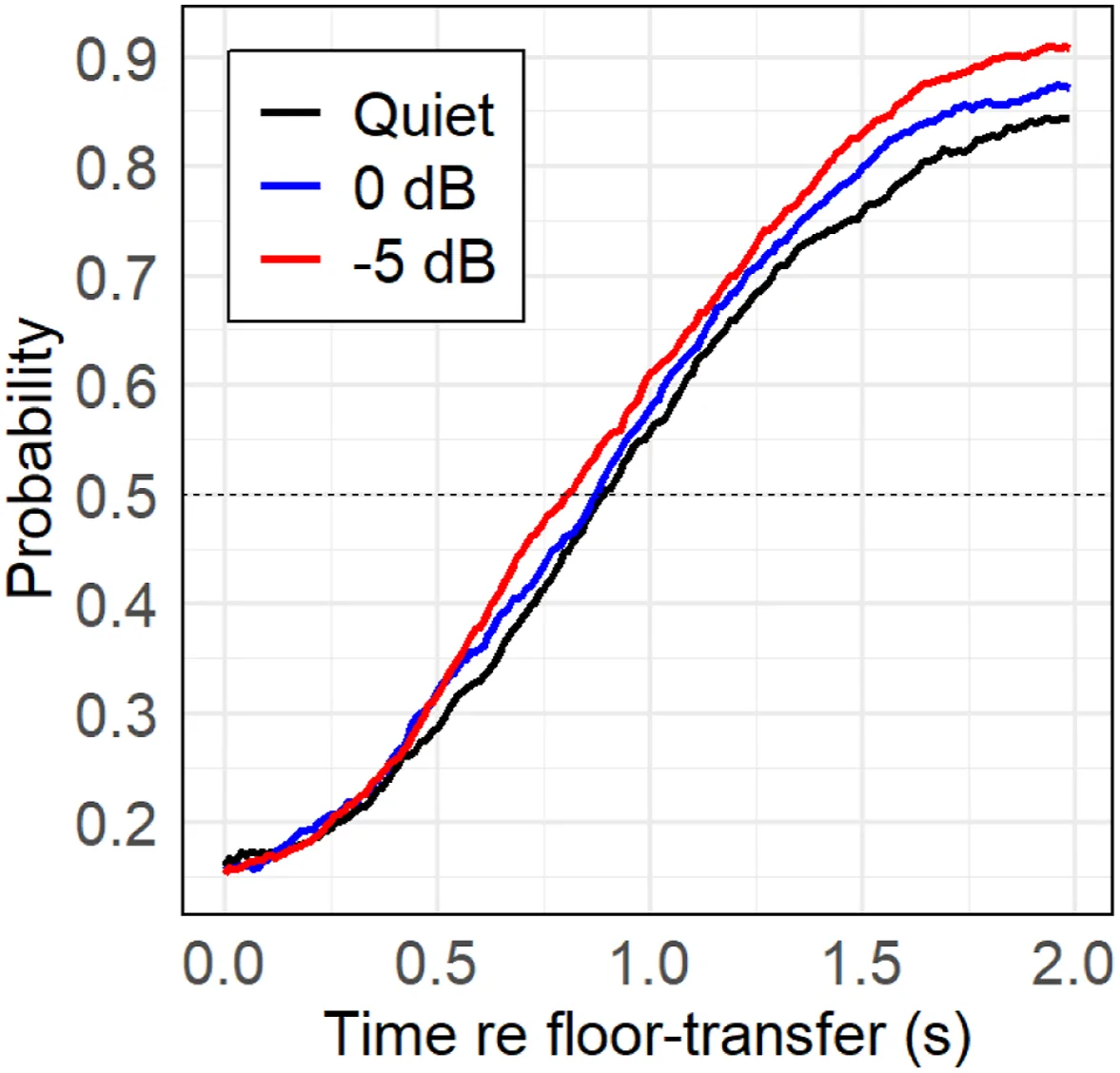
Probability that a participant looked toward the new talker as a function of time relative to the floor transition and SNR as a parameter

In the five head-turn parameters that were derived from the individually fitted logistic functions, only the head-turn delay (τ_50_) and the maximum probability (*p*_max_) parameter revealed significant differences, with the quiet condition exhibiting significantly lower *p*_max_ (*p* < 0.05) and longer τ_50_ (*p* < 0.01) values than the -5dB SNR condition. All other comparisons did not reveal any significant effects. This finding was further confirmed for *p*_max_ using the bootstrapping approach from Seedorff et al. (2018), which compared the predicted (sigmoid) probability curves pairwise (between the three SNRs) to identify time instances at which the curves differ significantly between test conditions (see supplementary material, Figure 18). Similarly, the logistic regression model that was applied to the binary head-turn (time-series) data only revealed a significantly longer head-switch delay in the quiet condition when compared to the -5 dB SNR condition (*p* < 0.05).

### Associations With Listening Effort

When subjective listening-effort ratings were modeled as a function of SNR, hit rate, RT, head-switch delay, and maximum probability of looking at the new talker, only SNR was significantly associated with perceived listening effort (*p* < 0.001). This indicates that the behavioral outcome measures did not show independent associations with subjective listening effort beyond the effect of listening condition.

When RT was used as a behavioral measure of listening effort and modeled as a function of SNR, head-switch delay, and maximum probability of looking at the new talker, SNR (*p* < 0.001) and head-switch delay (*p* < 0.05) were both significantly associated with RT, whereas maximum probability of looking at the new talker was not significant. This indicates that both less favorable SNRs and longer head-switch delays were associated with longer response times.

## Discussion

### Sensitivity of the Proposed Speech Comprehension Test

Sensitivity is an essential feature of any speech test (e.g., Brand and Kollmeier (2002)), and outcomes need to be sensitive to the relevant differences between test participants and between test conditions within the same participant. Sensitivity to *participant* differences means that the test can be used to evaluate an individual’s speech comprehension ability. The measure can then be used to assess the factors that affect speech comprehension ability, such as hearing loss and cognitive decline. Sensitivity to the differences between *test conditions* can index each participant’s speech comprehension ability in different task difficulties. This measure can then be used to assess the benefit of hearing interventions, such as hearing devices and other treatments. For both participant and test conditions, the sensitivity of an assessment is limited by the unexplained variance within the measured data. Understanding this variance and its underlying sources is therefore important for interpreting outcomes.

In the present study, we evaluated listening effort ratings, hit rate, and RT as outcome measures within a newly developed speech comprehension test. Recall that subjective listening effort ratings were used to indicate an individual’s perceived listening effort, hit rate was used to indicate how precisely an individual can follow a real-life conversation, and RT may provide additional information on higher-level processing, such as semantic and cognitive processing speed. Although there are underlying differences among these three measures, they all showed significant differences across the different task difficulty levels, i.e., SNR (see Figure 7). Therefore, increasing task difficulty can indeed increase the participants’ perceived listening effort, decrease their hit rate, and increase their RT.

However, these differences were observed only when the data were averaged across a relatively large number of participants and test conditions (i.e., repetitions), and across widely spaced difficulty levels (i.e., quiet, 0 dB SNR, and -5 dB SNR). Hence, although no systematic sensitivity analysis was conducted in this study, the efficiency of the developed test appears limited, at least in comparison to that of a (clinical) sentence test (e.g., Brand and Kollmeier., 2002). This limitation is not unexpected, given the various sources that might have contributed to the unexplained variance. These include participant-related differences in auditory, visual, and cognitive processing, as well as variability in the speech materials, as discussed further below and illustrated in the supplementary material (Figure 12-17).

Part of the unexplained variance likely arose from differences between participants. Although all participants were young adults with normal hearing and self-reported English as a first language, they likely differed in their familiarity with Australian-accented speech, semantic knowledge, and attentional strategies. Accent familiarity can affect speech perception in noise, particularly when listeners must adapt to unfamiliar speech patterns, and vocabulary knowledge may influence how effectively contextual information is used (Banks et al., 2015). Likewise, differences in semantic knowledge may affect how readily target words are linked to the designated topic (Lupyan & Lewis, 2019), and differences in attentional engagement may further influence comprehension performance in demanding listening conditions (Bhandari et al., 2022). Such factors may help explain why listening-effort ratings, hit rate, and RT became more variable across participants as SNR decreased, even though all three measures showed the expected overall effect of listening difficulty.

A second major source of unexplained variance likely came from the speech materials. Because the test was developed from spontaneous, unscripted audiovisual conversations, the resulting snippets naturally varied not only in topic and content, but also in spectral characteristics, speaking rate, and conversational dynamics. Such variability is ecologically valid, but it also increases unexplained variance relative to more tightly controlled clinical materials. Spontaneous speech contains reductions, disfluencies, and variable lexical realization that can affect speech processing and understanding (Ernestus & Warner, 2011; Mattys et al., 2012). In addition, the topics themselves likely differed in semantic complexity and familiarity, meaning that some snippets may have required less cognitive effort to follow than others even within the same nominal SNR condition.

The strength of association between target words and their assigned topic also likely varied across items. For example, ‘summer’ was strongly associated with the category ‘seasons of the year’, whereas ‘Western’ was less directly associated with ‘culture’ and may therefore have required greater reliance on contextual interpretation. Notably, the ‘seasons of the year’ topic may have been particularly straightforward, as it constituted a closed set of only four possible target words, whereas most other topics represented a more open set of potential target words without such a restriction. Similarly, some target words were more predictable from the local conversational context than others; for instance, ‘Italy’ within the category ‘location’ may have been easier to anticipate in a context-rich exchange than a less predictable item. Furthermore, because the task was audiovisual, some items may also have benefited from visible gestures or referential actions. For example, words such as ‘wrist’ and ‘knees’ belonged to the same semantic category but may not have been equally supported by visual cues (i.e., gestures).

Variability in the acoustic realization of the target words likely further contributed to the response-time variability. The materials included repeated lexical items across snippets, such as ‘minutes’, ‘toll’, and ‘Hong Kong’, as well as multiword expressions such as ‘Cross City Tunnel’ and ‘Swiss fondue cheese’. In spontaneous speech, such items are naturally heterogeneous in duration, clarity, loudness, prosodic context, and uniqueness point (i.e., the moment at which a word becomes uniquely identifiable). The RT in the present study was referenced to target-word onset, and therefore, these word-level differences may have introduced substantial variability even within the same listening condition. More generally, some target words produced floor effects even in quiet, whereas others remained close to ceiling across conditions, indicating that not all items provided comparable sensitivity. Taken together, these sources of material-related variance likely reduced the sensitivity of the present test, even though they also reflect the natural heterogeneity of real conversational speech.

### Head Movement in Multi-Talker Speech Comprehension

In this study, we analyzed participants’ head movements while listening to two-talker conversations. Examination of the head azimuth histograms (see Figure 8) showed that most participants generally oriented toward the active talker. Consistent with Lu et al. (2021), head rotations typically undershot the physical talker positions, indicating that visual localization was achieved through coordinated head and eye movements rather than head rotation alone. This pattern may suggest that participants primarily turned their heads to access visual information from the active talker while still maintaining some benefit from acoustic head shadow (Grange et al., 2018). Turning an ear toward the target would have provided a larger acoustic benefit, whereas fully orienting toward it would have provided the smallest benefit (and also put excessive strain on the participant’s neck).

The overall head azimuth angle distributions showed no noticeable differences across the three SNR conditions, indicating that participants predominantly directed their gaze toward the active talker regardless of task difficulty. This finding is consistent with participants’ post-experiment feedback, which confirmed their reliance on visual cues. However, there were three outliers who exhibited no significant head or eye movement and always focused on the front direction. Most likely the task was easy enough for them that they did not need to rely on any visual cues and preferred to only listen to the two talkers to focus better on the task, although they did not achieve significantly higher hit rates or shorter RTs compared to other participants. The dynamic head motion analysis around conversational floor transfers revealed that head-turn timing depended on listening conditions. Head-switch delays decreased with decreasing SNR, indicating earlier head turns in more challenging conditions. This effect was robust across multiple statistical analyses, including sigmoid function fitting, bootstrapping, and logistic regression, with a significant difference observed between the quiet and −5 dB SNR conditions. Taken together, these findings suggest that in more adverse listening environments, listeners initiate head movements earlier, possibly to anticipate upcoming speech and utilize visual information proactively.

This observation contradicts several previous studies reporting delayed gaze or head switches under more challenging listening conditions (e.g., Hidalgo et al. (2022) and Shiell et al. (2023)), suggesting that degraded auditory input may impair prediction of conversational turn transitions. This discrepancy likely reflects important methodological differences between the present and previous paradigms.

First, the present study employed a wide-field audiovisual display (±45°), requiring substantial head rotation to access visual speech cues. This setup may have encouraged proactive visual orienting in adverse conditions. In contrast, some prior studies constrained head movement or used front-facing displays, potentially reducing the need for proactive head orienting. Second, the present task involved comprehension monitoring rather than interactive turn-taking. In non-interactive settings, listeners are not constrained by the social pressure to continue looking at the current talker until it is relatively certain that the turn is ending, and may therefore adopt more anticipatory orienting strategies, particularly when auditory input is degraded. Third, the SNR range tested (quiet to −5 dB) may have been too easy for normal-hearing participants. Even in the most challenging condition (-5 dB SNR) the average listening-effort rating was only 3.8, where a rating of 4 corresponded to “moderate effort.” At lower SNRs, participants would likely need to engage greater cognitive resources, which could reduce processing speed when anticipating conversational floor transfers and consequently increase measured head-turn delays. Future research should therefore further investigate the combined effects of increased reliance on visual cues and higher cognitive demand at lower SNRs on head-turn behavior. Additionally, the maximum probability of looking at the new talker (*p*_*max*_) increased with decreasing SNR, indicating greater reliance on visual cues in more challenging conditions. The increased reliance on visual cues in more challenging conditions might also be the reason that listeners initiate their head movements earlier (in the included SNR conditions).

Although head-switch delays became shorter on average as SNR decreased, relatively longer head-switch delays were additionally associated with longer RTs after accounting for SNR in the second stage of our analysis. This suggests that across SNR conditions, listeners may turn their heads earlier in more difficult listening conditions as a strategy to make more use of visual information, whereas within a given listening condition, slower orienting toward the next talker may delay access to visual cues that support comprehension, and thereby delay understanding of the incoming speech. Thus, although the overall SNR effect on head-turn behavior in the present study differed from Hidalgo et al. (2022), the within-condition association is broadly consistent with their finding that more anticipatory orienting is linked to more efficient (i.e., faster) speech comprehension.

### Listening Effort and Speech Comprehension

Subjective ratings provide a direct measure of individuals’ perceived listening effort. In the present study, only SNR was significantly associated with perceived listening effort, while hit rate, RT, head-switch delay, and maximum probability of looking at the new talker did not show independent associations. This suggests that, in the present test, the behavioral outcome measures were more strongly influenced by listening condition than by subjective listening effort. Thus, these measures seem primarily informative as indicators of performance and orienting behavior, rather than listening effort.

Behavioral measures of listening effort are commonly desirable as they do not require explicit listener feedback and may be less susceptible to response bias than subjective measures (Holube et al., 2024; Schepker et al., 2016). However, RT, the behavioral measure of listening effort used in this study, was not significantly associated with subjective effort ratings. This casts some doubt on whether these two measures reflect the same underlying construct. Indeed, dissociation between subjective and behavioral measures of listening effort have been reported previously (Alhanbali et al., 2019; Strand et al., 2018) and it has been argued that, due to the impact of motivation on spoken language processing, single-task processing speed metrics may in fact be poor indicators of listening effort (Holt et al., 2020). The use of naturalistic speech material and a single-task design in the test may have further undermined the usefulness of RT as a metric for listening effort, limiting direct comparison with other more controlled sentence tests and dual-task paradigms (e.g., Holube et al., 2024; Picou & Ricketts, 2014) and introducing multiple alternative sources of RT variability. Accordingly, the significant association found between RT and head-switch delay may be better interpreted as a relationship between head-turn behavior and speech-comprehension efficiency, rather than between head-turn behavior and effort.

### Limitations and Outlook

This exploratory study successfully developed a new, realistic, multi-talker speech comprehension test. However, while the variability introduced by the realistic speech materials, stimuli, and outcome measures may reflect important real-world behavior, it also limited the sensitivity of the test for hearing assessment applications.

First, the hit rate of some target words was low even in the quiet condition, suggesting that most participants either did not recognize the words or did not associate them with the designated category. Such target words are considered unreliable items and likely reduce test sensitivity. Future studies should consider excluding these items to enhance test reliability.

Second, certain target words consistently reached ceiling performance across all test conditions, indicating that these words were too easy. This may have occurred because some words were highly predictable from the conversational context, strongly associated with the designated semantic category, or supported by salient visual cues. Future studies should consider replacing these overly easy items or testing them at lower SNR to improve sensitivity.

Third, relatively stationary and artificial speech babble was used as background noise to minimize noise-related variance on the evaluated outcome measures. Future studies should incorporate additional ecological factors such as realistic background noise, room reverberation, and distracting talkers to better reflect real-life listening environments. Controlling these factors may help identify which aspects of complex listening environments contribute most to listening difficulties for individual listeners.

Fourth, the study did not include physiological measurements of listening effort. While subjective self-ratings provide valuable insights into perceived effort, these measures are inherently limited by individual task interpretation, scale usage, and other subjective biases. Similarly, behavioral measures of listening effort, such as RT, can pose interpretation issues as they may not directly reflect cognitive resource use. Physiological measures offer more direct and objective assessments of listening effort. Although pupillometry (via the Tobii glasses) was not feasible in our test environment due to the lighting variation from the videos, future studies could consider alternative methods such as electroencephalography, functional near-infrared spectroscopy, and autonomic nervous system measures such as skin conductance and heart rate.

Fifth, analyzing gaze switch delays posed significant challenges in the current study. Previous studies examining gaze switch timing used paradigms that either restricted the participants from moving their heads or were designed so they did not need to move their heads (Hidalgo et al., 2022, 2024; Shiell et al., 2023). These studies presented video recordings on a screen that was within the participants’ front gaze view. In contrast, the current study presented recordings on a 200° screen with talkers located at ±45°, requiring participants to move both their heads and eyes to access relevant visual information. However, rapid eye movements often prevented the micro-cameras in the eye-tracking glasses from accurately capturing gaze data, leading to significant data loss precisely during conversational transitions – the critical events for analyzing gaze switch delays. Future studies should either restrict the spatial separation between talkers, although that would compromise ecological validity, or employ more sensitive eye-tracking technology.

Sixth, the elicited vocal-effort levels in the recordings were not systematically matched to the playback SNR conditions. This reflects the staged development of the test: the present study first aimed to establish its feasibility and sensitivity under controlled noise conditions, whereas future work will focus on increasing ecological realism. In particular, future studies will combine the audiovisual conversation materials with realistic Ambisonic background-noise recordings (e.g., Weisser et al., 2019), and match vocal effort levels to the corresponding acoustic environment.

Finally, the current study provides valuable normative data for assessing different populations and hearing interventions. Future work should extend the test to listeners with hearing loss to evaluate hearing aid benefit in complex, real-world listening situations. Ultimately, integrating this test into virtual reality technologies could broaden access across the research community and enhance its applicability in clinical settings.

## Supplemental Material

Supplemental Material - Development and Evaluation of a Realistic Audio-Visual Multi-Talker Speech Comprehension Test Supplemental Material for Development and Evaluation of a Realistic Audio-Visual Multi-Talker Speech Comprehension Test by Xinyu Guo, Rebecca Holt, Kelly Miles, Ronny Kurniawan Ibrahim, Stefan Raufer, Peter Derleth, Joerg M. Buchholz in Trends in Hearing.

## Appendix

### Topics and Target Words

Table 1 shows snippet IDs, corresponding topics, and target words. The number in the bracket refers to the occurrence of target words in the corresponding snippet. For example, ‘Minutes (3)’ means that the word ‘Minutes’ occurred three times in snippet A_2_1.

**Table 1.** Selection of Topics and Target Words

Snippets	Topic	Target words
A_2_1	Unit of time	Minutes (3)
A_2_2	Unit of time	Hour (1); Minutes (1)
A_2_3	Driving	Cross city tunnel (1); Toll (2); Drive (1)
A_2_4	Expenses	Toll (2); Rent (1)
A_2_5	Driving	Traffic (1); Indicate (1); Indicating (3)
A_2_6	Driving	Left lane (1); Middle lane (1); Right lane (1); Left (1)
A_2_7	Relatives/people	Lady (1); Kids (1); Husband (1)
A_2_8	Unit of distance or speed	110kms (1); 150-160kms (1)
A_2_9	Personality traits	Annoying (1); Aggressive (2)
A_3_1	Movie genres	Scary movies (1); Comedic zombie movies (1)
A_3_2	Water activities	Catamaran (1); Snorkeling (1)
A_3_3	Relatives/people	Grandmother (1); Mother (1)
A_4_1	Location	Hong Kong (1); Italy (2); Switzerland (1)
A_4_2	Snowsport	Skiing (1); Ski (2)
A_4_3	Location	London (4); Berlin (4)
A_4_4	Famous people	Anne Frank (1); Van Gogh (1)
A_4_5	Location	Argentina (1); Europe (1)
A_4_6	Relatives/people	Cousins (1); Family (2)
A_4_7	Alpine	Mountain (1); Peak (1)
A_5_1	Location	Australia (1); Hong Kong (2)
A_5_2	Music	CDs (1); Album (1); Radio (1)
A_5_3	Music	Gangster rap (1); Hip hop (1); MJ (1)
A_6_1	Entertainment	Song (1); Film (1)
A_6_2	Entertainment	Songs (2); Movies (1)
A_6_3	Movies	Frozen (1); The Little Mermaid (2); Beauty and the Beast (1)
A_6_4	Movies	Pocahontas (1); Bambi (2); The Little Mermaid (1)
A_7_1	Physical activities	Jumping (3); Jump (1); Walking (1); Crawling (1)
A_7_2	Seasons of the year	Summer (3)
A_7_3	Seasons of the year	Autumn (1); Summer (1); Winter (1); Spring (1)
A_8_1	Personality traits	Arrogance (1); Rude (1)
A_8_2	Modes of transportation	Car (2)
A_8_3	Places you drive	Street (1); Streets (1); Roads (1)
A_9_1	Relatives/people	Baby (2); Child (1)
A_9_2	Feelings	Laughing (2); Funny (1)
A_10_1	Food and cuisine	Dumplings (1); Swiss fondue cheese (1)
A_11_1	Christmas	Santa Claus (1); Santa (1)
A_11_2	Celebration	Gifts (1); Presents (1)
A_11_3	Animals	Pet (1); Turtle (1)
A_11_4	Items of clothing	Sandals (2)
A_12_1	Physical activity	Sport (2); Baseball (1)
A_12_2	Body parts	Wrist (1); Knees (2)
A_12_3	Numbers	Two (1); 30’s (1)
A_13_1	Theatre	International cinema (1); Director (1)
A_14_1	Travel	Airport (1); Road trip (1)
A_14_2	Entertainment	Movie (1); Film (1)
A_14_3	Food and cuisine	Breakfast (2); Brunch (1)
A_14_4	Food and cuisine	Paddle pops (1); Chocolate paddle pops (1)
A_14_5	Food and cuisine	Soft-serve (1); McDonalds (3); Cheeseburger (1)
A_15_1	Entertainment	VHS Videos (1); DVD (1); VHS tape (1)
A_15_2	Movies and entertainment	Warner Brothers (1); Batman (1); Pretty Woman (1)
A_15_3	Art	Watercolor artist (1); Paintings (1)
A_16_1	Culture	Chinese (1); Western (1)
A_17_1	Location	Mongolia (2); Europe (1)
A_18_1	Gaming consoles	PlayStation (1); PC (1); Nintendo (2)
A_18_2	Entertainment	Books (2); Read (3)
A_18_3	Activities/sports	Exercise (1); Running (1); Gym (1); Pilates (1); Yoga (1)
A_19_1	Humor	Laugh (1); Comedy (1)
A_19_2	Bugs	Cockroach (3)
