# Supplementary material for: Development and Evaluation of a Realistic Audio-Visual Multi-Talker Speech Comprehension Test
Source: Trends Hear. 2026 Sep 17;30:23312165261489985. doi: 10.1177/23312165261489985 (PMC13724885; doi:10.1177/23312165261489985)
Supplement: Supplementary file 1 [file sj-pdf-1-tia-10.1177_23312165261489985.pdf]

Supplementary

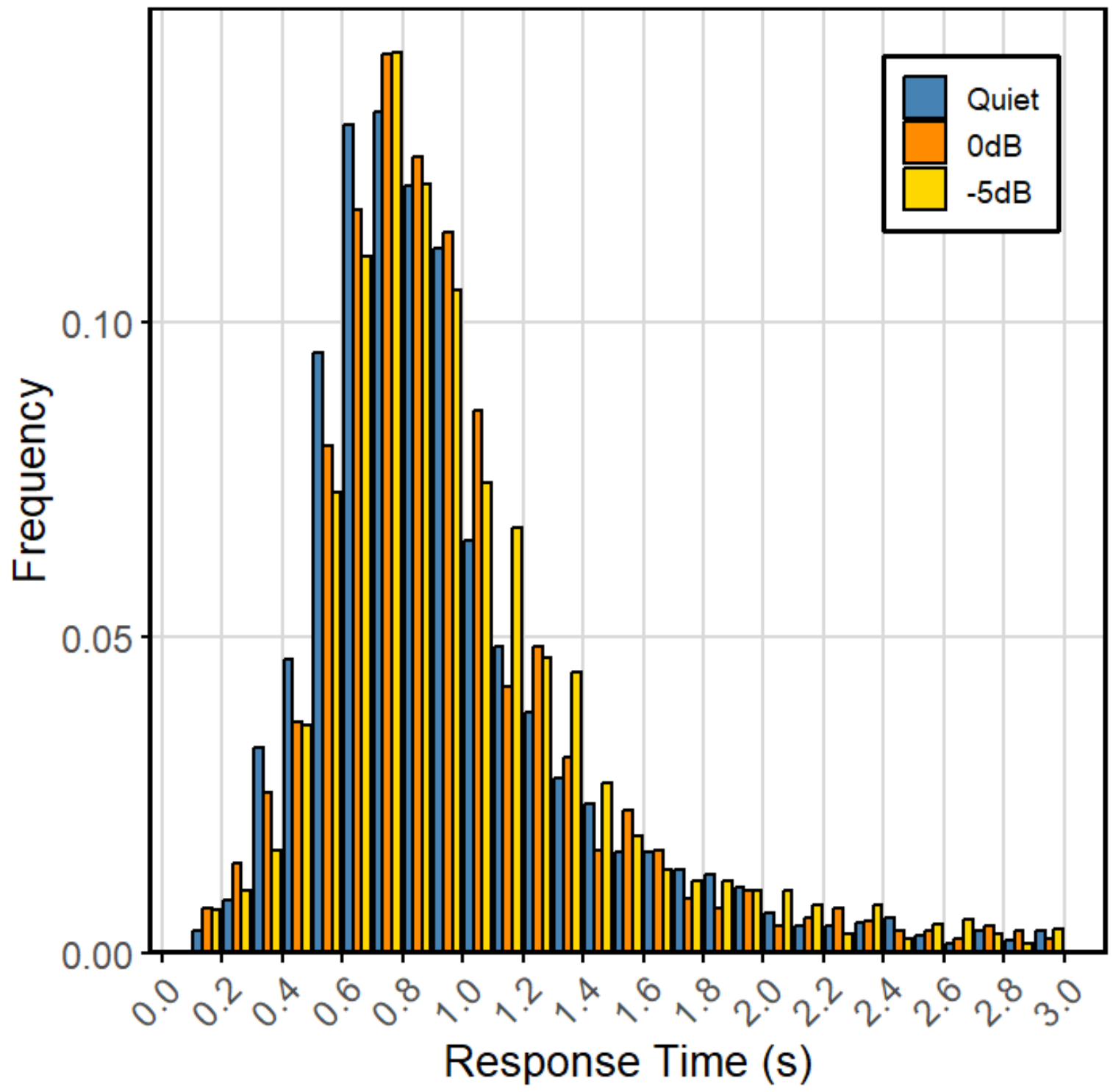

Figure 10. Histogram of RT.

Figure 10 shows histogram of the RTs. To capture the main distribution and realistic range of RTs, the RT data were restricted to values between 0.3s to 2s to remove potential outliers from analysis.

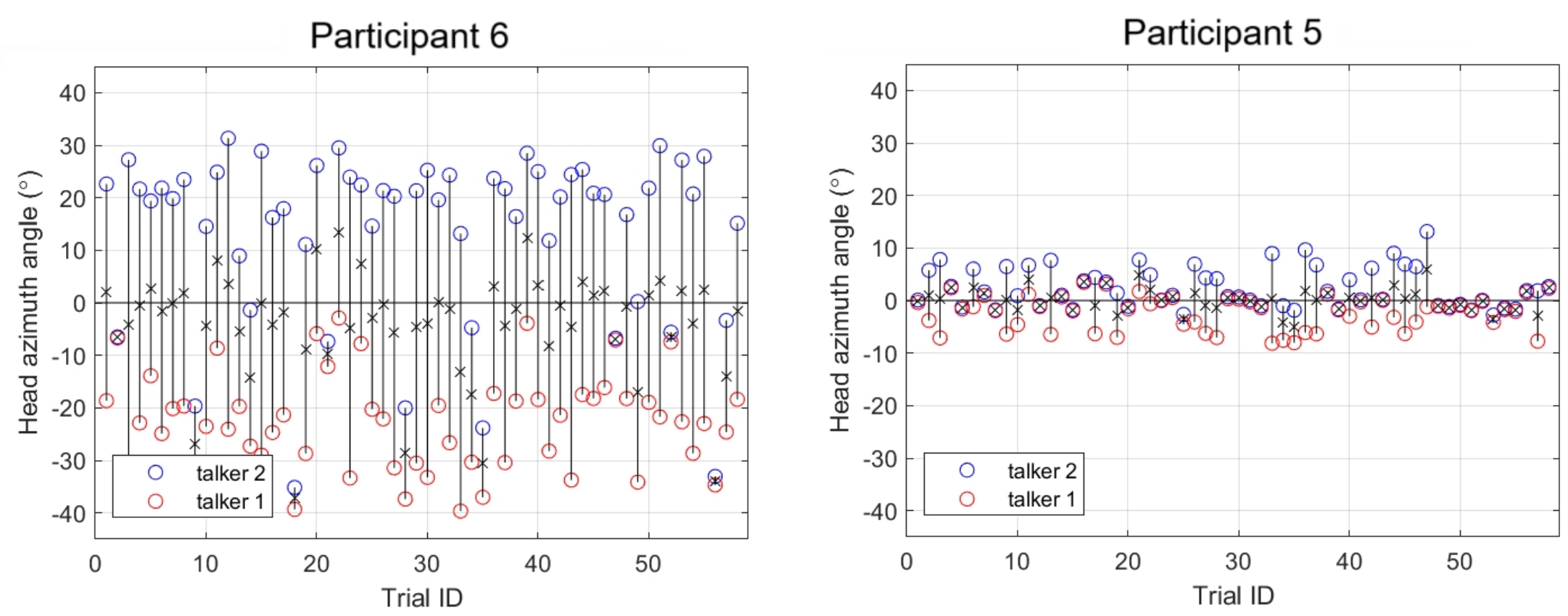

Figure 11. Estimated centroid locations (using k-means clustering) of the head azimuth angles for target talker 1 (red circles) and target talker 2 (blue circles) as a function of test trials for two example participants. Crosses show the midpoints between the two centroids.

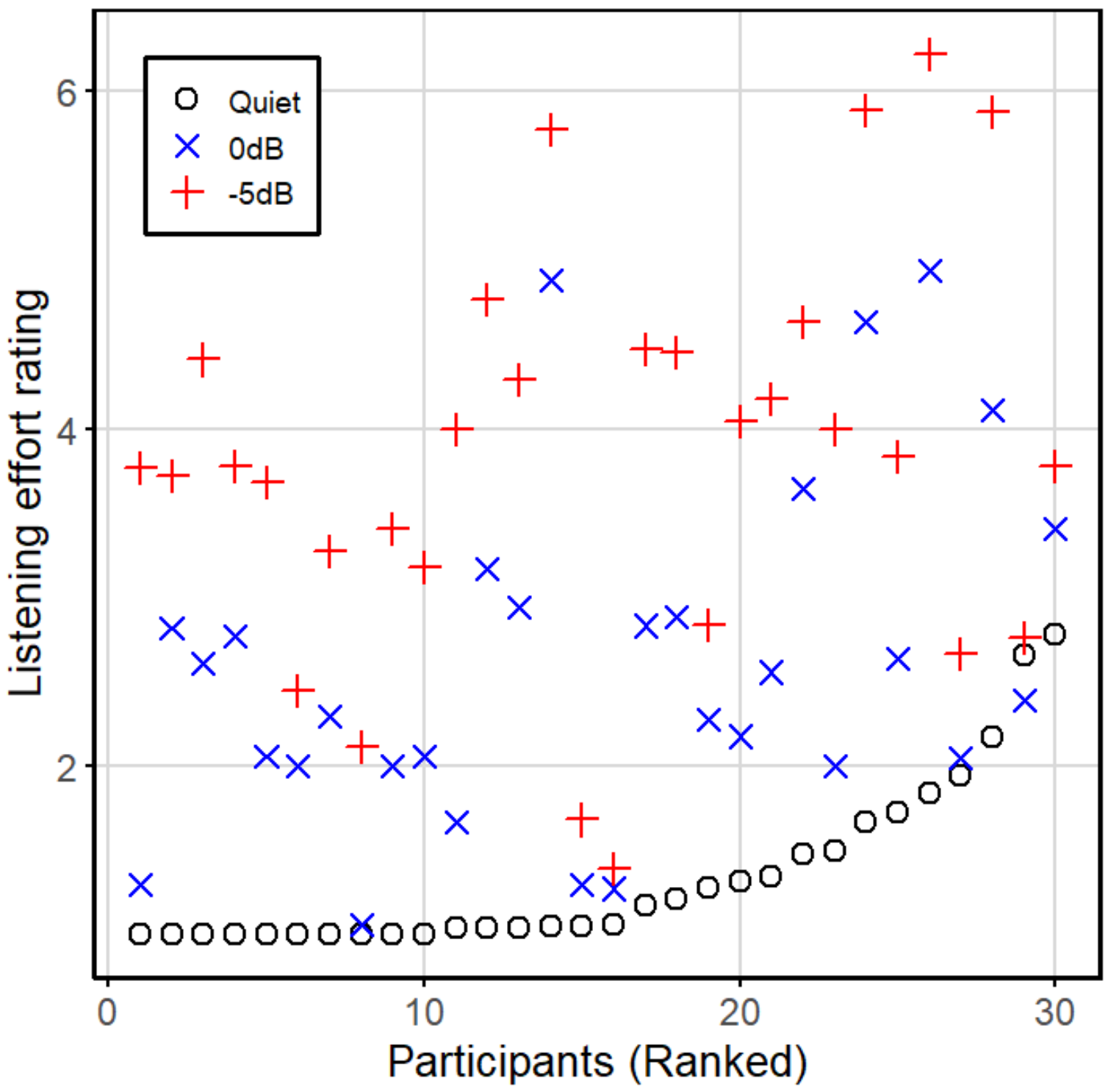

Figure 12. Ranked listening effort rating as a function of 30 participants.

Figure 12 illustrates the variance of the effort rating by showing average values across the 54 conversation snippets as a function of 30 participants and SNR as a parameter (see legend). The participants were ordered (ranked) according to ratings in the quiet condition.

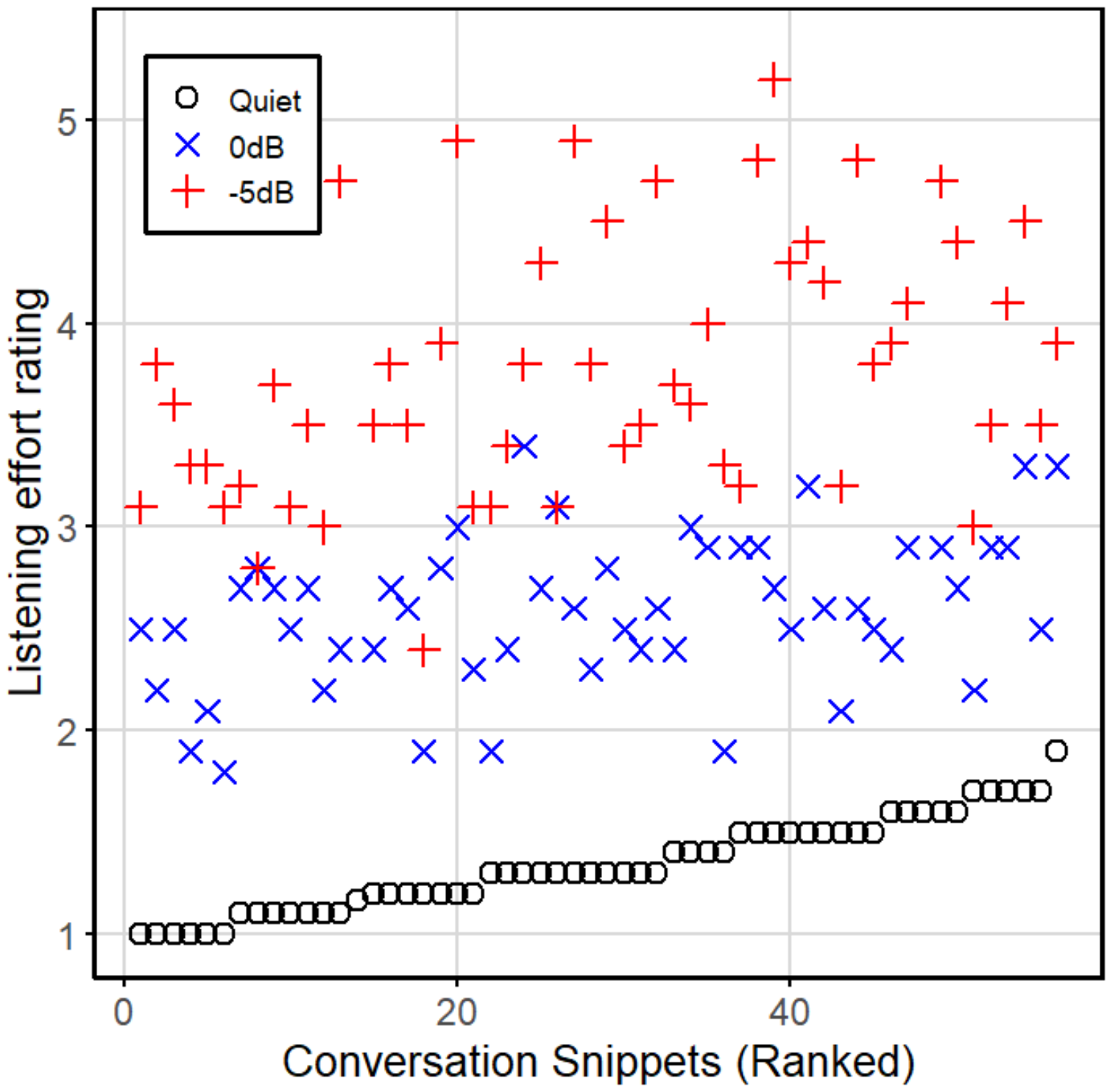

Figure 13. Ranked listening effort rating as a function of 54 conversation snippets.

Figure 13 illustrates the variance of the effort rating by showing average values across the 30 participants as a function of the 54 conversation snippets and SNR as a parameter (see legend). The conversation snippets were ordered (ranked) according to their ratings in the quiet condition.

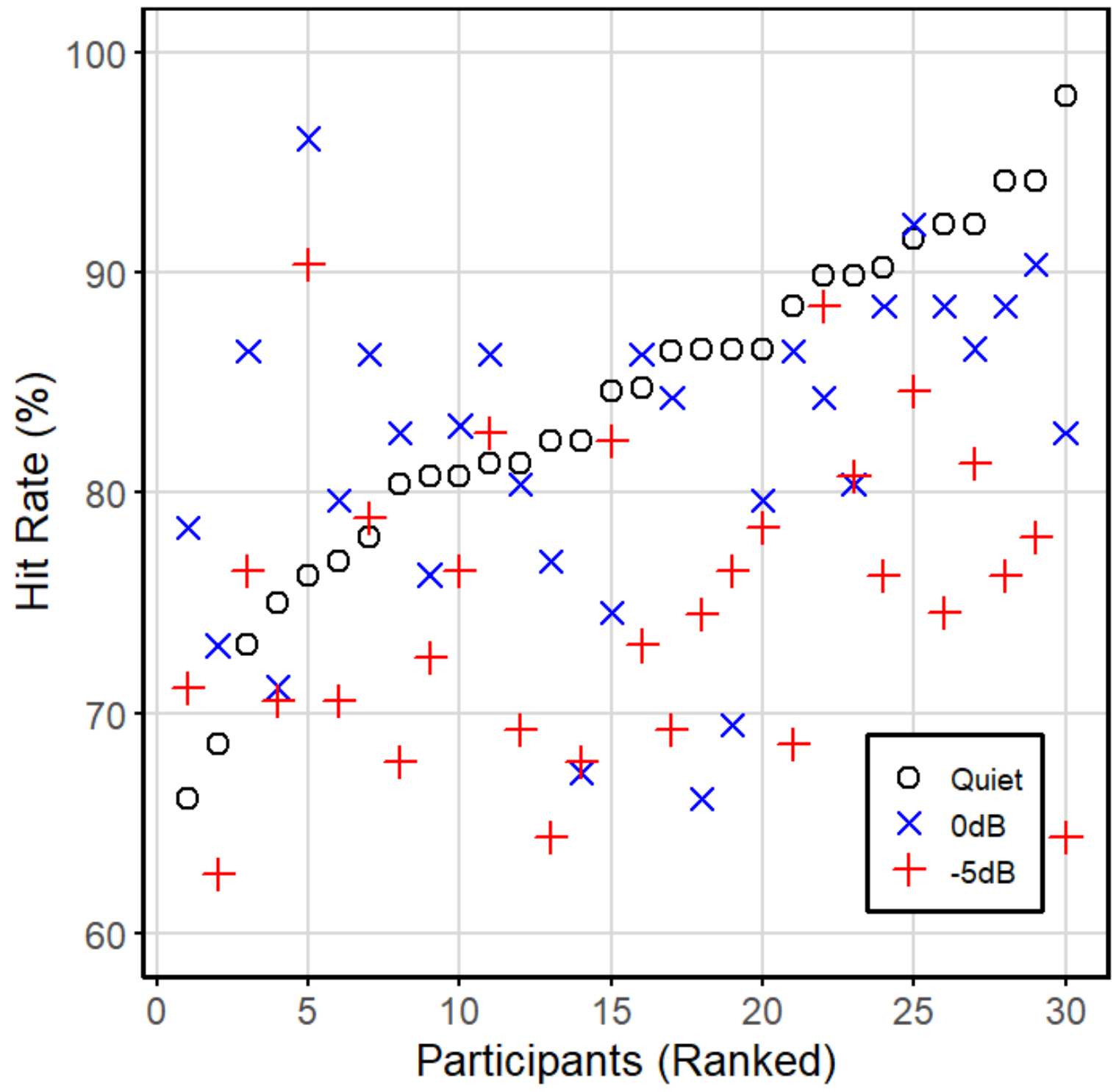

Figure 14. Ranked hit rate as a function of 30 participants.

Figure 14 illustrates the variance of the hit rate by showing average values across the 162 target words as a function of the 30 participants and SNR as a parameter (see legend). The participants were ordered (ranked) according to their hit rate in the quiet condition.

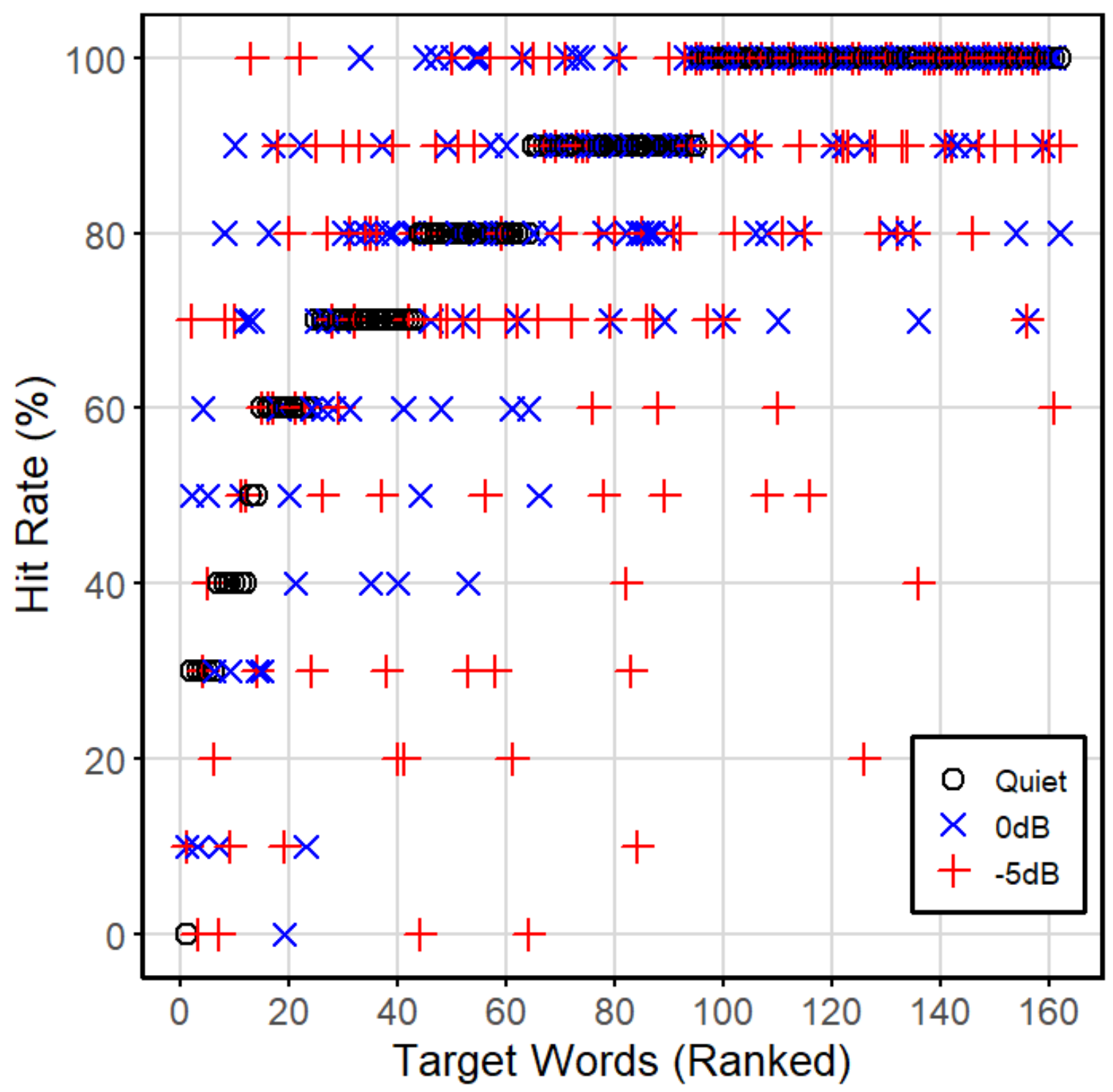

Figure 15. Ranked hit rate as a function of 162 target words.

Figure 15 illustrates the variance of the hit rate by showing average values across the 30 participants as a function of the 162 target words and SNR as a parameter (see legend). The target words were ordered (ranked) according to their hit rate in the quiet condition.

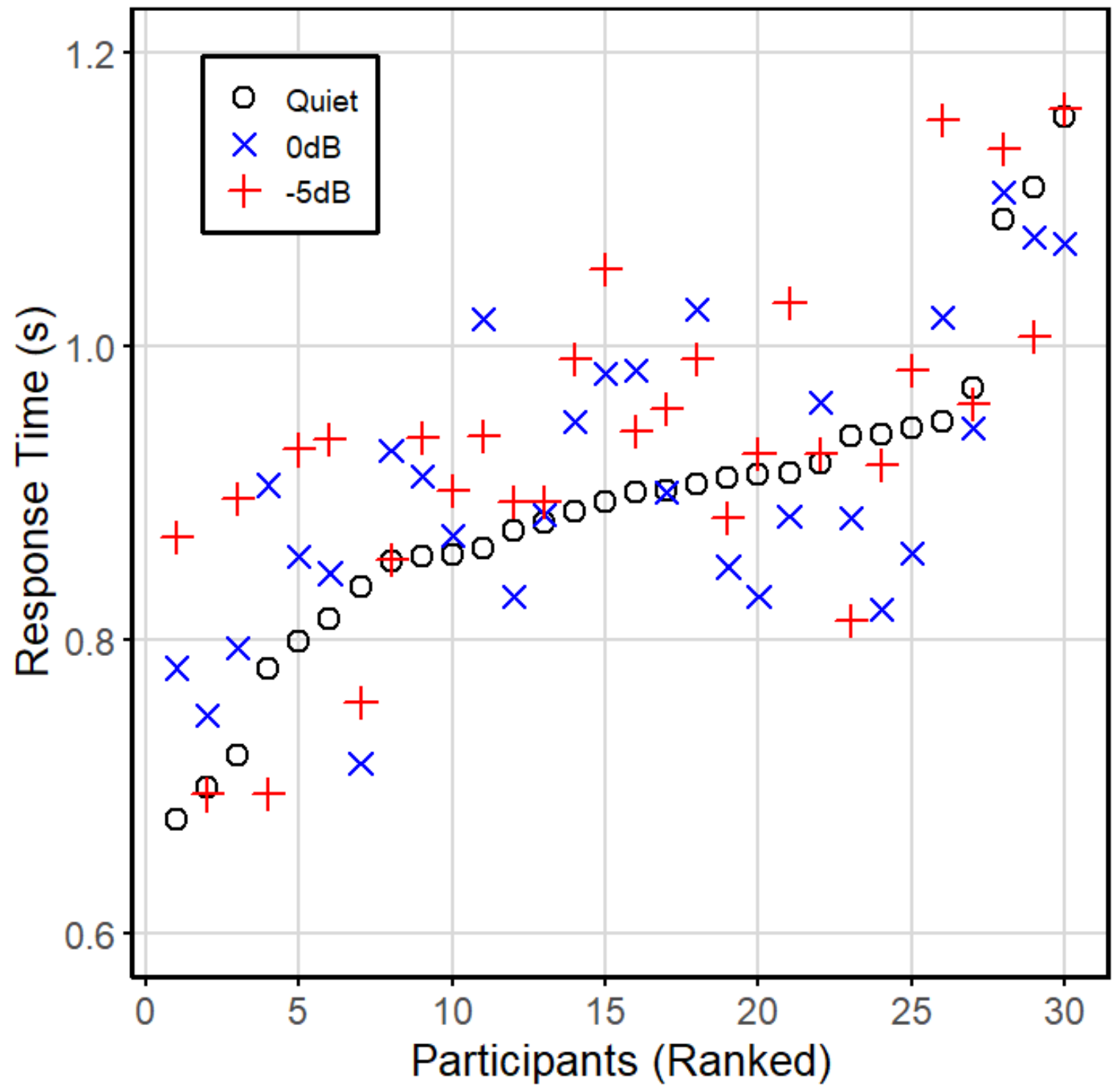

Figure 16. Ranked RTs as a function of 30 participants.

Figure 16 illustrates the variance of the RT by showing average values across the 162 target words as a function of 30 participants and SNR as a parameter (see legend). The participants were ordered (ranked) according to RTs in the quiet condition.

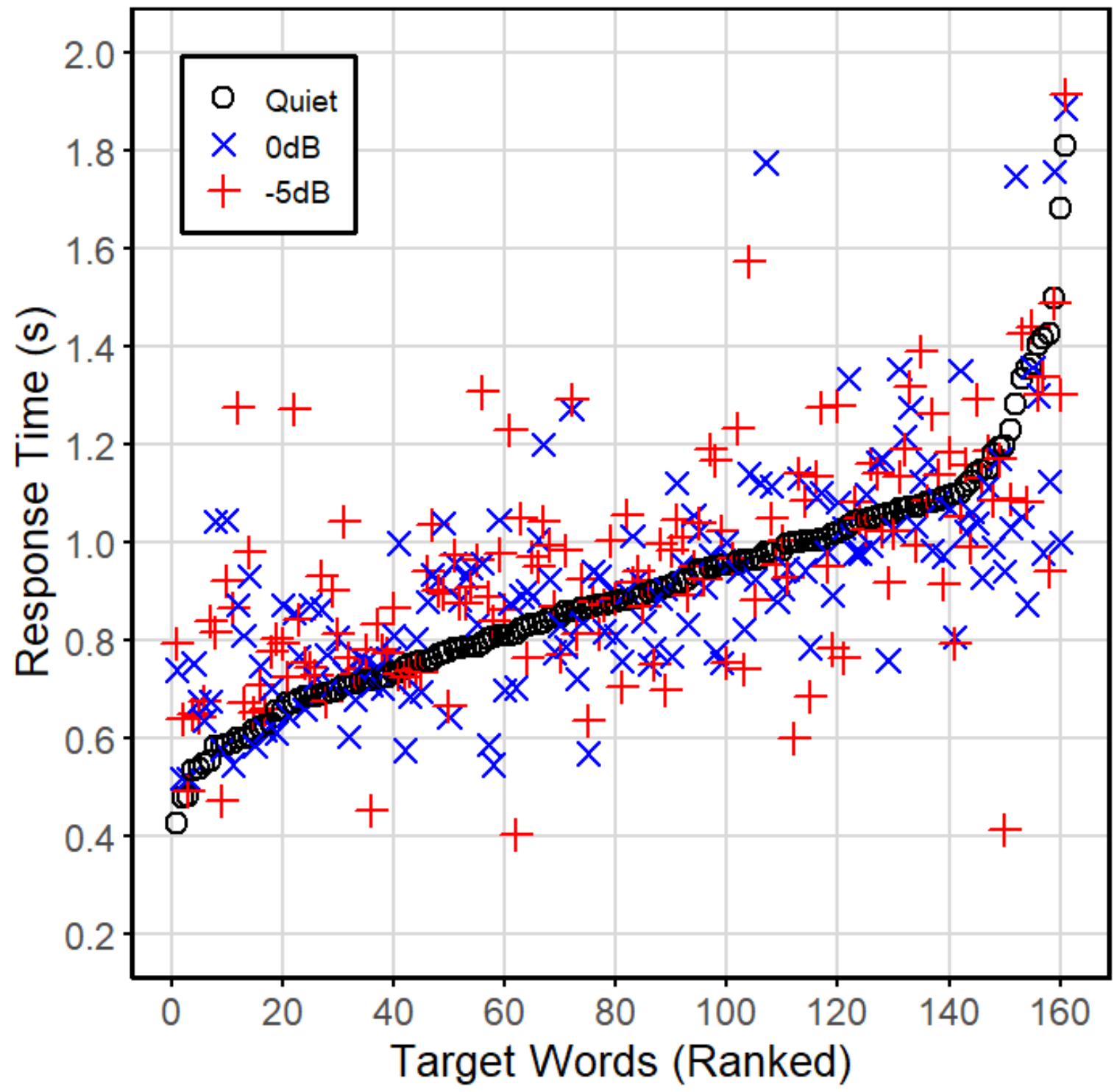

Figure 17. Ranked RTs as a function of the 162 target words.

Figure 17 illustrates the variance of the RT by showing average values across the 30 participants as a function of the 162 target words and SNR as a parameter (see legend). The target words were ordered (ranked) according to their RTs in the quiet condition.

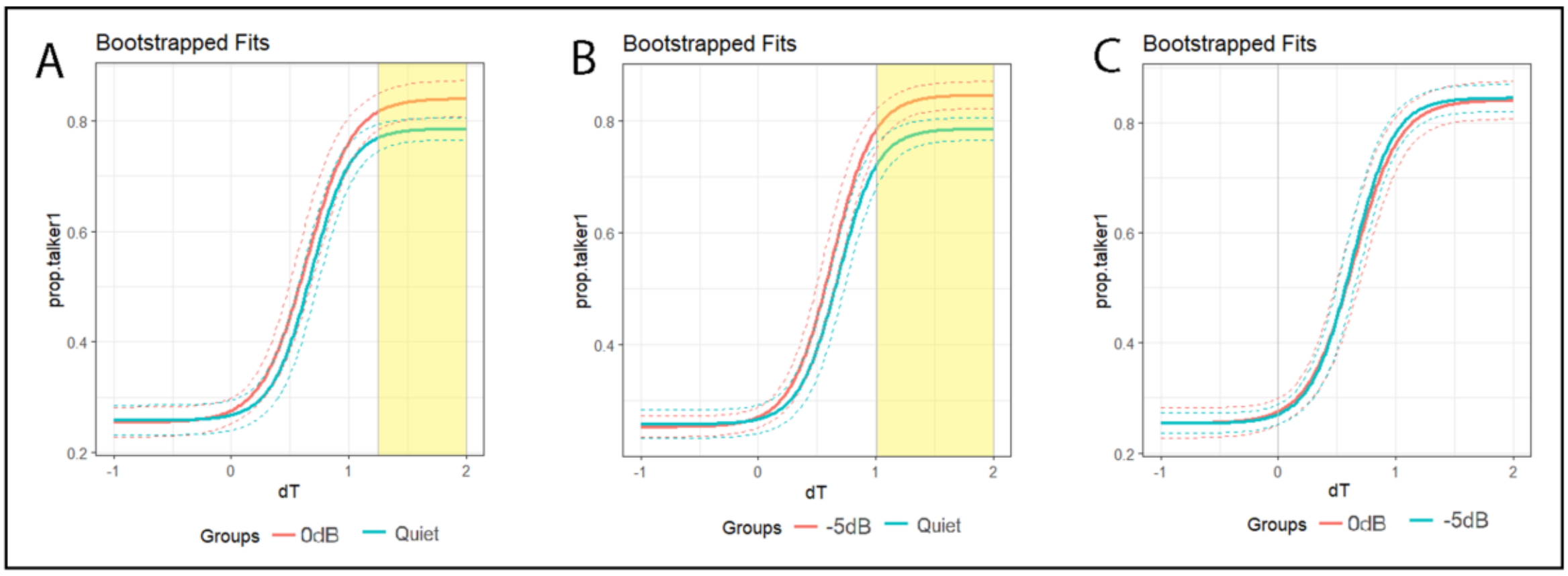

Figure 18. Bootstrapping comparison between different conditions. (a) Quiet (blue) vs. 0 dB SNR (red). (b) Quiet (blue) vs. -5 dB SNR (red). (c) -5 dB SNR (blue) vs. 0 dB SNR (red).

Figure 18 compared the predicted (sigmoid) probability curves pairwise (between the three SNRs) to identify time instances at which the curves differ significantly between test conditions. Time windows with significant differences between conditions shown in yellow.
